# A high-throughput screen of real-time ATP levels in individual cells reveals mechanisms of energy failure

**DOI:** 10.1371/journal.pbio.2004624

**Published:** 2018-08-27

**Authors:** Bryce A. Mendelsohn, Neal K. Bennett, Maxwell A. Darch, Katharine Yu, Mai K. Nguyen, Daniela Pucciarelli, Maxine Nelson, Max A. Horlbeck, Luke A. Gilbert, William Hyun, Martin Kampmann, Jean L. Nakamura, Ken Nakamura

**Affiliations:** 1 Gladstone Institute of Neurological Disease, San Francisco, California, United States of America; 2 Department of Pediatrics, University of California, San Francisco, California, United States of America; 3 Department of Radiation Oncology, University of California, San Francisco, California, United States of America; 4 Graduate Program in Biomedical Sciences, University of California, San Francisco, California, United States of America; 5 Department of Cellular and Molecular Pharmacology, University of California, San Francisco, California, United States of America; 6 Department of Urology, University of California, San Francisco, California, United States of America; 7 Helen Diller Family Comprehensive Cancer Center, University of California, San Francisco, California, United States of America; 8 Department of Laboratory Medicine, University of California, San Francisco, California, United States of America; 9 Department of Biochemistry and Biophysics and Institute for Neurodegenerative Diseases, University of California, San Francisco, California, United States of America; 10 Chan Zuckerberg Biohub, San Francisco, California, United States of America; 11 Department of Neurology, University of California, San Francisco, California, United States of America; 12 Graduate Program in Neuroscience, University of California, San Francisco, California, United States of America; University of Washington, United States of America

## Abstract

Insufficient or dysregulated energy metabolism may underlie diverse inherited and degenerative diseases, cancer, and even aging itself. ATP is the central energy carrier in cells, but critical pathways for regulating ATP levels are not systematically understood. We combined a pooled clustered regularly interspaced short palindromic repeats interference (CRISPRi) library enriched for mitochondrial genes, a fluorescent biosensor, and fluorescence-activated cell sorting (FACS) in a high-throughput genetic screen to assay ATP concentrations in live human cells. We identified genes not known to be involved in energy metabolism. Most mitochondrial ribosomal proteins are essential in maintaining ATP levels under respiratory conditions, and impaired respiration predicts poor growth. We also identified genes for which coenzyme Q10 (CoQ10) supplementation rescued ATP deficits caused by knockdown. These included CoQ10 biosynthetic genes associated with human disease and a subset of genes not linked to CoQ10 biosynthesis, indicating that increasing CoQ10 can preserve ATP in specific genetic contexts. This screening paradigm reveals mechanisms of metabolic control and genetic defects responsive to energy-based therapies.

## Author summary

There is abundant evidence that insufficient energy, or energy failure, contributes to the pathophysiology of many inherited and degenerative diseases, cancer, and aging. However, we understand little about how cellular energy levels are regulated. To begin to address this gap, we developed a screening approach that uses a fluorescent biosensor to measure relative levels of ATP in individual cells and used this approach to identify those genes that are most essential to maintaining energy levels. We screened approximately 2,200 genes and found that mitochondrial ribosomal proteins are particularly critical in maintaining energy levels and enabling cell growth. We also used this approach to identify genetic targets that could be amenable to an energy-based therapy via supplementation with the mitochondrial cofactor coenzyme Q10 (CoQ10). These included CoQ10 biosynthetic genes associated with human disease as well as a subset of genes not previously linked to CoQ10 biosynthesis. Our screening paradigm thus reveals mechanisms by which cellular energy levels are controlled. It can also be used to identify genetic defects that will be responsive to energy-based therapies. Application of this approach may additionally enable powerful screens for other key metabolites to further advance a genome-scale understanding of the regulation of metabolism.

## Introduction

ATP is the key energy-carrying molecule in all cells, and failure to maintain adequate ATP levels may be critical in many diseases, ranging from mitochondrial disorders to cancer and neurodegeneration [1–3]. Despite the importance of ATP, we understand little about the contributions of genes and pathways that maintain its levels and how they are regulated.

Genes that modulate ATP levels may be therapeutic targets for diseases of energy failure or candidates for novel inborn errors of metabolism. Finding these genes would provide insight into how energy failure contributes to disease. However, that analysis has been limited by a lack of tools to screen the genome at high throughput for modifiers of ATP levels. One innovative study assayed ATP with arrayed adherent cells and identified genes that, when knocked down by RNA interference (RNAi), decreased or increased cellular ATP content [4]. Although an important starting point, the study was limited by a small number of library short hairpin RNAs (shRNAs) per gene, and it assayed total ATP normalized to DNA content and so could not differentiate changes in cell size or cell cycle from ATP concentration.

The primary sources of ATP are mitochondrial oxidative phosphorylation and cytoplasmic glycolysis. However, which pathway predominates and the genetic control points are determined by poorly understood factors. In addition, functional assays are needed to match specific energy-based therapies to disorders that are likely to benefit from these therapies. For example, cofactor therapies, such as coenzyme Q10 (CoQ10) supplementation, have been used therapeutically in disorders of CoQ10 biosynthesis [5], but with limited efficacy in other mitochondrial disorders [6].

Genetically encoded fluorescent biosensors specifically report the level of their target metabolite in living cells, including ATP [7], NADH [8], lactate, and glucose [9]. Measuring metabolites in individual cells instead of cell lysates has produced important observations of the temporal, spatial, and subcellular regulation of cellular metabolism. However, high-throughput technologies, such as flow cytometry, have yet to be combined with fluorescent biosensors to detect metabolites in screens and other large studies.

Here, we report a novel screening paradigm to identify genes that maintain cellular ATP levels. We combined fluorescence resonance energy transfer (FRET) and fluorescence-activated cell sorting (FACS) to measure ATP in single living cells. This screen shows that FRET and FACS can be used to screen a metabolite based on the real-time level and to provide valuable insight into key pathways and therapeutic strategies to maintain ATP in human cells.

## Results

### Optimizing an ATP-FRET sensor for FACS

FRET describes the process by which photons emitted from an excited fluorophore are transferred to a second fluorophore with an efficiency dependent on the physical proximity of those fluorophores. FRET-based biosensors operate on the principle that the binding of a metabolite to a specific protein domain tethered to 2 fluorophores changes the sensor’s confirmation and the distance and orientation of the fluorophores, thus fluorescently reporting the concentration of that metabolite. FRET-based biosensors for metabolites have been used with fluorescence microscopy, but flow cytometry also detects FRET [10]. To our knowledge, sorting cells based on the real-time levels of a metabolite has not been done.

**Fig 1:**
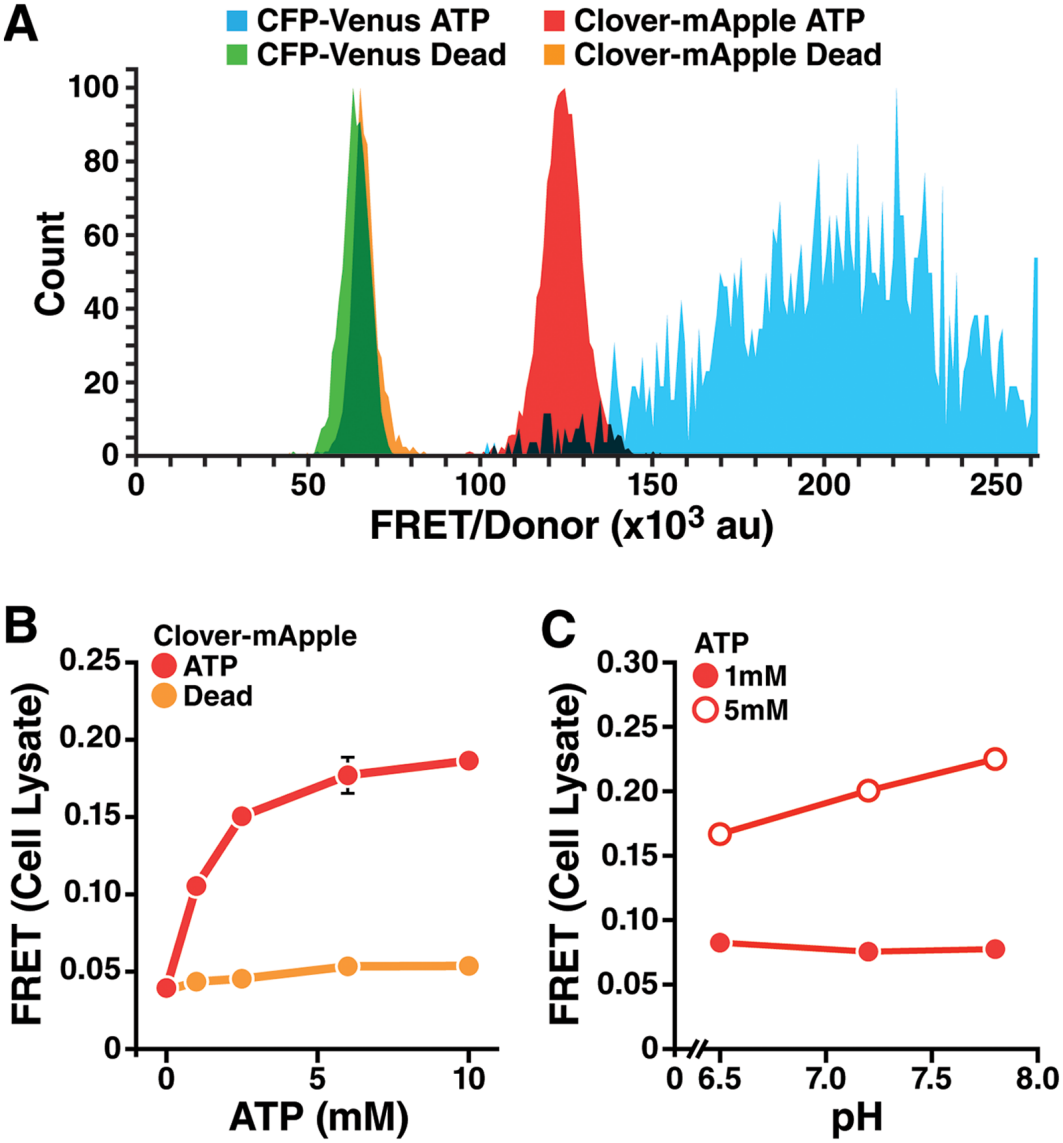
A FRET-based ATP sensor compatible with flow cytometry. (A) FRET/donor distributions of K562 cells stably expressing CFP-Venus ATP FRET sensor (AT1.03^YEMK^, blue) with CFP (donor) and Venus (acceptor) [7] or the modified Clover-mApple ATP sensor (red) with Clover (donor) and mApple (acceptor). CFP-Venus Dead (green) and Clover-mApple Dead (orange) are the corresponding ATP-binding–deficient mutant sensors. Tracings show the distribution of 2,500–4,500 cells. The experiment was repeated twice with similar results. Clover-mApple ATP produces a narrower distribution of FRET/donor values than CFP-Venus ATP, and the dead sensors had similar distributions. (B) FRET signal of COS cell lysates expressing the Clover-mApple ATP or Dead sensor, incubated with increasing ATP. The Clover-mApple ATP sensor was responsive to ATP concentrations up to approximately 6 mM, much higher than the CFP-Venus ATP sensor (S1H Fig). Data show mean ± SD (bars obscured by points); *n* = 3 wells/group. (C) Cell lysates of COS cells expressing the Clover-mApple-ATP sensor were incubated in buffer SH, with increasing pH and/or ATP. Data show mean ± SD; *n* = 4 wells/group. Increasing pH did not affect the ATP FRET signal at 1 mM. At 5 mM ATP, there was a small but significant association of increasing pH and FRET (*p* < 0.0001 by one-way ANOVA). Further information about this figure can be found in S1 Data. CFP, cyan fluorescent protein; COS, CV-1 (simian) in origin, and carrying the SV40 genetic material; FRET, fluorescence resonance energy transfer; mVenus, monomeric Venus.

We started with an established sensor with modified cyan fluorescent protein (CFP) and monomeric Venus (mVenus) (AT1.03^YEMK^, CFP-Venus ATP) separated by an ATP-binding domain [7,11]. This sensor shows ATP-dependent changes in FRET at physiological ATP concentrations and is resistant to pH changes [7,11]. We found that it also reports ATP levels by flow cytometry as it had a higher FRET/donor signal than a mutant sensor with no ATP affinity (AT^R122K/R126K^, CFP-Venus Dead) [7] (Fig 1A and S1A Fig). Furthermore, drugs blocking oxidative phosphorylation and glycolysis (to stop all ATP synthesis) reduced the FRET signal (S1B Fig). Unfortunately, the FRET signal varied based on expression level of the sensor, limiting its ability to detect differences in ATP between cells (S1C Fig, see also Materials and methods). This limitation would confound population-based measurements needed for a screen. We hypothesized that this artifact is due to the relative dimness of CFP and the poor overlap of CFP with available FACS laser excitation wavelengths (405 and 488 nm, with E_max_ of CFP approximately 433 nm), causing reduced detection of FRET at low expression levels.

To improve the sensor, we substituted fluorophores using the same interposed ATP-binding domain [12]. We selected Clover (donor) and mApple (acceptor) for their brightness, spectral overlap, and compatibility with standard flow cytometer laser and emission filter wavelengths [13,14]. The Clover-mApple sensor had the expected cytosolic localization in cells (S1D Fig) and produced a narrow distribution of FRET that reported ATP levels (Fig 1A). The FRET signal was decreased by substituting the dead mutant that disrupts ATP binding (Clover-mApple Dead, S1E Fig) or by adding drugs to inhibit oxidative phosphorylation and glycolysis (S1F Fig). The signal was independent of expression of the sensor (S1G Fig). In vitro, the dynamic range of the CFP-Venus ATP sensor approached saturation approximately 3 mM ATP (S1H Fig). Because cytosolic ATP levels are estimated in the low mM range [11,15], the CFP-Venus sensor may be saturated at baseline ATP levels [7], which precludes detection of increased ATP and likely reduces sensitivity to small decreases in ATP. Substituting green and red fluorophores for CFP and Venus can reduce the affinity of the sensor for ATP [12], and the Clover-mApple ATP sensor FRET saturated close to 6-mM ATP (Fig 1B), indicating an improved capacity to detect higher ATP levels versus the CFP-Venus sensor. The Dead sensor showed little response to exogenous ATP (Fig 1B) and can thus be a control to identify artifactual FRET changes independent of ATP binding. The Clover-mApple sensor showed limited sensitivity to pH similar to the CFP-Venus sensor [7] (Fig 1C). Because of the improved brightness, compatibility with standard flow cytometry lasers, dynamic range, and lack of FRET variation based on sensor expression level compared with the CFP-Venus sensor, the Clover-mApple ATP and Dead sensors were used for the high-throughput measurement of ATP in individual cells.

### CRISPRi screen for determinants of cellular ATP level

**Fig 2:**
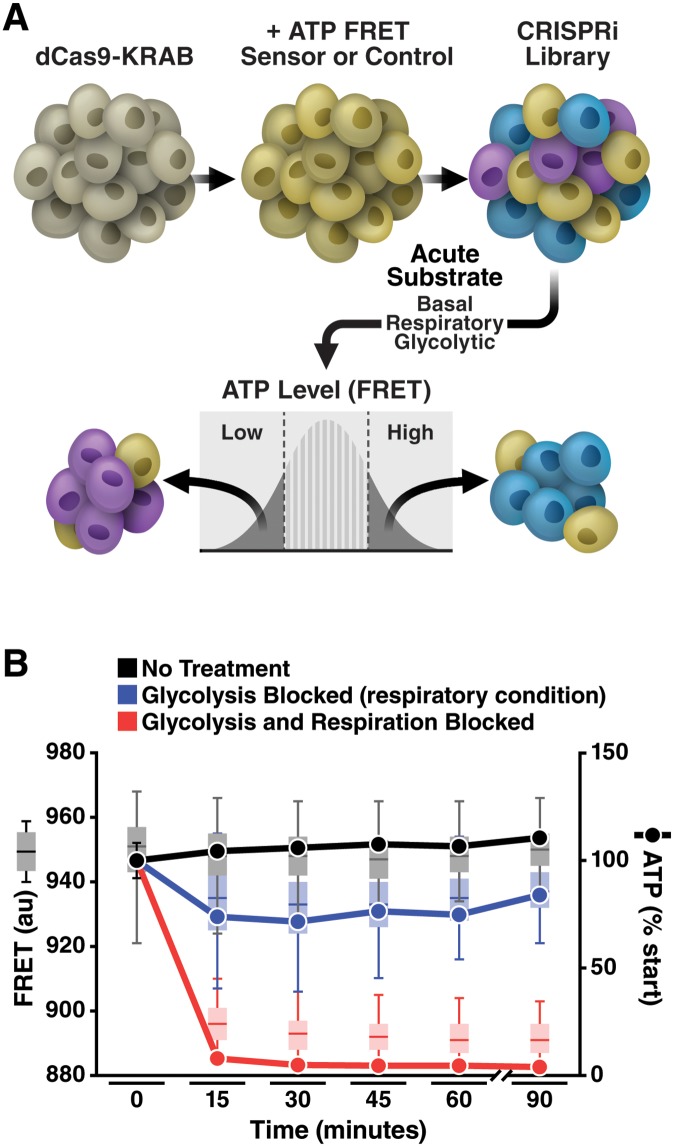
Single-cell detection and sorting based on ATP content. (A) Schema illustrating ATP FACS sorting protocol. K562 cells were transduced to stably express the dCas9-KRAB and then the ATP FRET sensor (or Dead sensor), and a small population was selected for dual expression by FACS. These cells were then expanded and transduced with a CRISPRi sgRNA lentiviral library so that each cell individually expressed one of approximately 28,000 sgRNAs. Cells were selected for expression of the sgRNA by puromycin for 4–5 days, allowed to recover from puromycin, and then exposed to substrate and drug conditions forcing ATP synthesis from glycolysis only (oligomycin and glucose) or oxidative phosphorylation only (2DG and pyruvate) 30 minutes prior to sorting. Cells were then sorted for 60 minutes. Cells in the lowest and highest ATP quartiles were isolated by flow cytometry, and the abundance of each sgRNA was quantified in the high- and low-ATP quartiles by deep sequencing. (B) Time course of ATP decline after incubation of cells with a glycolytic inhibitor (10 mM 2DG) and pyruvate (10 mM) to force reliance on respiration for ATP (“respiratory” conditions) or when both respiration and glycolysis were blocked (10 mM 2DG and 5 μM oligomycin) to prevent all ATP production. ATP was measured both by FRET with the Clover-mApple ATP sensor using flow cytometry (left y-axis, box and whisker plots; line = median; box = 25th–75th percentile; whisker = 5th–95th percentile) and by luciferase assay (right y-axis, lines, error bars ± SD not visible beyond the points). Both show similar relative extents and rates of ATP decline. Respiratory conditions produced a partial drop in ATP level that is stable for at least 75 minutes as measured by both FACS and luciferase (*p* < 0.0001 versus both control and blocked glycolysis/respiration groups at each time point after start by two-way ANOVA with Tukey multiple comparisons test; *n* = 6,024–21,295 cells/group for FACS; *n* = 3 samples/group for luciferase). The FRET experiments were repeated with similar results (S2A Fig). Further information about this figure can be found in S1 Data. 2DG, 2-deoxyglucose; CRISPRi, clustered regularly interspaced short palindromic repeats interference; dCas9, dead CRISPR-associated protein 9; FACS, fluorescence-activated cell sorting; FRET, fluorescence resonance energy transfer; KRAB, Kruppel-associated box; sgRNA, single guide RNA.

Clustered regularly interspaced short palindromic repeats interference (CRISPRi) technology allows the knockdown of individual genes in a large population of cells. We generated K562 cells that express a dead CRISPR-associated protein 9 (dCas9)-Kruppel-associated box (KRAB) fusion protein, which allows CRISPRi to reduce gene expression [16,17], followed by the Clover-mApple ATP FRET sensor (hereafter, ATP FRET sensor) or the Dead sensor (Fig 2A). We then transduced these cells with pooled lentivirus containing approximately 28,000 single guide RNAs (sgRNAs) targeting 2,231 genes in a mitochondrial gene-enriched subgenome library. The library contained 10 distinct sgRNAs per gene and 1,400 non-targeting control sgRNAs. This many controls could not be tested by lower-throughput methods.

ATP can be derived from oxidative phosphorylation, glycolysis, or both. To know the source of ATP, the screen was conducted 3 times, wherein cells were acutely exposed to different drug and substrate conditions that forced ATP synthesis to derive from (1) oxidative phosphorylation only, (2) glycolysis only, or (3) from both. We forced cells to use oxidative phosphorylation—the “respiratory” condition—by incubating cells with high-dose 2-deoxyglucose (2DG; 10 mM) to inhibit glycolysis and 10 mM pyruvate to support aerobic respiration without glucose. To limit homeostatic mechanisms or cell death, cells were switched to these treatments only about 30 minutes before sorting, leaving cells little time to adapt.

In the respiratory condition, ATP levels declined modestly, as assessed by luciferase and flow cytometry FRET (Fig 2B, blue line and box and whiskers, respectively, and S2A Fig an independent repetition), showing that FRET is in the dynamic range of the sensor during screening and that cellular energy was acutely stressed but not depleted. The relative changes in ATP as measured by FRET or luciferase were similar, indicating that our ATP-FRET sensor reliably reflects ATP levels. Adding 5 μM oligomycin to block oxidative phosphorylation in combination with high-dose 2DG resulted in rapid and complete loss of ATP (Fig 2B, red line [luciferase] and box and whiskers [FRET]), confirming that, in 2DG alone, ATP was actively being made and that all detected ATP was from mitochondrial oxidative phosphorylation. The Dead sensor showed only a minimal reduction in FRET upon inhibiting glycolysis and oxidative phosphorylation (S2B Fig), although ATP levels measured by luciferase decreased as expected (S2C Fig), indicating that the Dead sensor is a suitable control for genetic manipulations that alter FRET independent of ATP.

To restrict ATP production to glycolysis only—the “glycolytic” condition—cells were exposed to 2 mM glucose and 5 μM oligomycin (S2D Fig). A low dose of 2DG (3 mM) was also added to the glycolytic condition to reduce ATP from baseline because ATP levels did not acutely decrease in K562 cells with oligomycin alone in glucose. As with the respiratory condition, reducing ATP below baseline levels during cell sorting was necessary to ensure that ATP levels were within the dynamic range of the sensor and to confirm that cellular energy was acutely stressed. Increasing 2DG to 10 mM in the glycolytic condition resulted in loss of ATP (S2C Fig), confirming that detected ATP was derived from glycolysis. Finally, we screened a “basal” condition with PBS, 5 mM pyruvate, 10 mM glucose, and no inhibitory drugs.

Because FACS occurs over time, we noted the stability of ATP levels in each metabolic condition over the timeframe (30–60 minutes post treatment) needed to sort cells. For cells in respiratory or glycolytic conditions, ATP levels assessed by the ATP FRET sensor were stable for 90 minutes as detected by luciferase or flow cytometry FRET (Fig 2B and S2D Fig). To conduct the screen, cells expressing the ATP FRET sensor, dCas9-KRAB, and a single sgRNA were exposed to acute metabolic conditions as above, and the highest and lowest quartiles of ATP as reported by the ATP FRET sensor were collected (Fig 2A). To control for ATP-independent changes in FRET (e.g., from unexpected effects of the sgRNA on the synthesis or turnover of the sensor fluorophores), we conducted a parallel screen with the Dead FRET sensor. We collected approximately 200 cells per sgRNA (average of 10 sgRNAs/gene, approximately 2,000 cells/gene); with this method, about 1,000 genes can be screened and sorted per hour. Each collected quartile was deep sequenced, and the number of each sgRNA in each FRET quartile was determined by counting the sequencing reads for that sgRNA in that quartile [17]. We generated a phenotype (fold-enrichment in the high- versus low-FRET quartile) for each sgRNA.

The screen was repeated 3 times for each of the 3 metabolic contexts (respiratory, glycolytic, basal) with the ATP FRET sensor, and twice with the Dead sensor in each context. The phenotype of each individual sgRNAs was averaged over the repetitions. The final gene phenotype was reported as the average of the 3 sgRNAs with the strongest phenotype per gene. We examined the raw phenotypes for separate sgRNAs for each repetition and found that the reproducibility of each sgRNA compared with non-targeting sgRNAs was good (S3 Fig, select genes, S1 Table for all average phenotypes, S2 Table for all individual sgRNA phenotypes for the respiratory condition for all 3 repetitions).

### ATP-critical pathways depend on metabolic context

**Fig 3:**
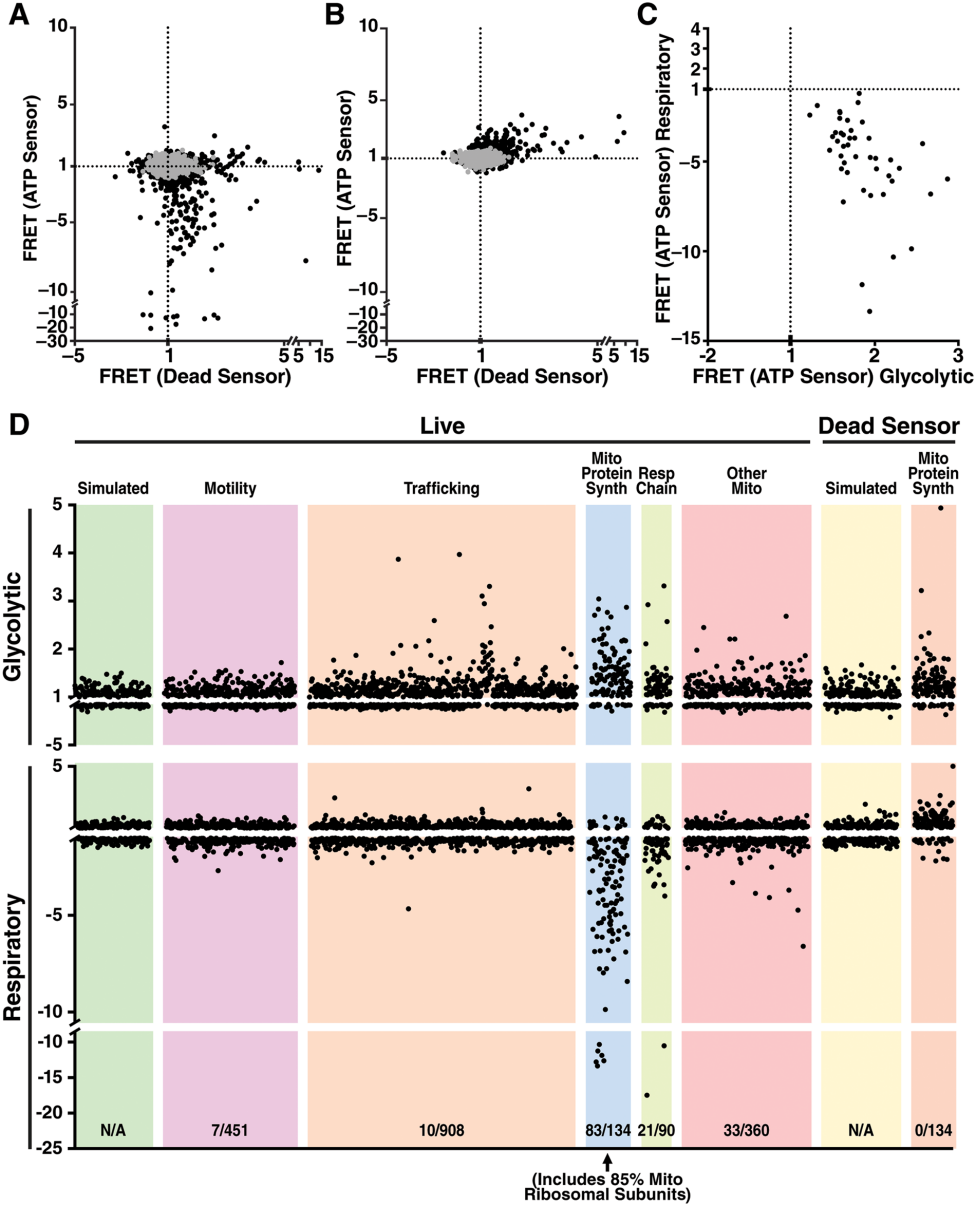
Critical role for mitochondrial protein synthesis genes in maintaining ATP under respiratory but not glycolytic conditions. (A, B) Cells expressing an sgRNA from a mitochondrial-gene–enriched CRISPRi subgenome library with 10 sgRNAs/gene and either the ATP FRET sensor or Dead FRET sensor were placed in either (A) respiratory (10 mM 2DG and 10 mM pyruvate) or (B) glycolytic (5 μM oligomycin, 2 mM glucose and low-dose [3 mM] 2DG) conditions, and the cells were sorted by ATP concentration. Abundance of each sgRNA in the high- and low-FRET fractions was determined by deep sequencing, and the relative enrichment of each sgRNA in the high- versus low-ATP fraction determined. Black dots are individual gene phenotypes based on the average of the 3 sgRNAs with the largest phenotypes, averaged per sgRNA over 3 independent experiments. Grey dots are simulated genes consisting of random combinations of non-targeting sgRNAs. For each gene, the y-axis shows fold-enrichment in high versus low-ATP fractions for cells expressing the ATP FRET sensor, and the x-axis shows the corresponding fold-enrichment in high versus low-ATP fractions with the Dead FRET sensor. (C) Comparison of the ATP FRET phenotypes (3 strongest sgRNAs averaged over 3 repetitions) for genes considered “hits” in both respiratory and glycolytic conditions (see S3 Table). x-axis = glycolytic condition; y-axis = respiratory condition. Note that no genes that decreased ATP in the respiratory condition when knocked down also decreased ATP in the glycolytic condition, and knockdown of many genes decreasing ATP in the respiratory condition protected ATP in the glycolytic condition. (D) Fold-enrichment of individual genes in the high- versus low-ATP fractions (by FRET, y-axis) for cells expressing the ATP FRET sensor, with each point as the mean enrichment of the 3 sgRNAs with the largest fold-enrichment magnitudes, for glycolytic and respiratory conditions (also shown in panel A and B). Genes are grouped by general function, including motility, trafficking, “mito protein synth,” “resp chain,” and “other mito.” The glycolytic condition is on top and the respiratory condition below. Also presented are simulated genes of non-targeting sgRNAs (simulated) and mitochondrial protein synthesis gene knockdown by the Dead FRET sensor. Further information about this figure can be found in S1 Table. 2DG, 2-deoxyglucose; CRISPRi, clustered regularly interspaced short palindromic repeats interference; FRET, fluorescence resonance energy transfer; mito protein synth, mitochondrial protein synthesis; other mito, other mitochondrial functions; resp chain, respiratory chain; sgRNA, single guide RNA.

To assess the specificity of our screen for true hits that alter ATP levels, we considered the distribution of non-targeting sgRNAs. To simulate the analysis for the active CRISPRi sgRNAs, we tested random combinations of 10 sgRNAs from among the 1,400 non-targeting sgRNAs, each combination representing a simulated gene, and graphed the average of the 3 sgRNAs with the strongest phenotypes for each simulated gene (Fig 3A and 3B grey dots). The phenotypes of these simulated genes were tightly clustered around 1 (equal distribution in the high- and low-FRET fractions), thus representing the “noise” in the screen. A gene was defined as a “hit” in the respiratory condition if there were 2 or more sgRNAs yielding phenotypes >2 SDs beyond the mean phenotype of the non-targeting sgRNAs, averaged over the 3 independent repetitions. Using these criteria, none of the simulated genes was identified as a hit under any of the 3 metabolic conditions (fold-enrichment in high versus low-ATP fractions all approximately 1) (Fig 3 and S1, S2 Tables), suggesting the specificity of the screen for detecting sgRNAs that affect gene expression. Consistent with this, many genes targeted by active sgRNAs were strikingly enriched in the low-ATP fraction when sorted under respiratory conditions (Fig 3A).

To control for artifactual hits that might impact the FRET signal independent of the ATP level, the entire sublibrary was also rescreened 2 times with the Dead sensor, and the same analysis was performed (Fig 3A and 3B). Potential hits that similarly altered FRET with the Dead sensor were removed from analysis. Under respiratory conditions, most hits that decreased the FRET signal with the ATP sensor had little effect on the FRET signal of the Dead sensor (Fig 3A), indicating that they are true low-ATP hits. However, occasional genes did influence the FRET signal of the Dead sensor, especially under glycolytic conditions (Fig 3B), indicating that ATP-independent effects can also impact the FRET signal and underscoring the importance of a dead mutant control. An analogous control is not available for luciferase-based assays.

To determine the effect of metabolic context of gene knockdown on ATP levels, we compared the phenotypes of each gene in respiratory and glycolytic conditions. While few genes decreased the ATP FRET signal in glycolytic conditions, knockdown of some genes caused small increases in the ATP FRET signal in glycolytic conditions (Fig 3B, S3 Table). Among these, some gene-targeting sgRNAs also increased the FRET of the Dead sensor (Fig 3B). These artifacts were due to the effect of gene knockdown rather than general effects of CRISPRi because artifacts were not observed with non-targeting sgRNAs. In the glycolytic condition, 65 of 144 (45.1%) of FRET-increasing hits were removed because they also increased FRET in the Dead sensor control. ATP-independent artifacts seemed to be pathway specific: 20 of 29 (60.0%) of cytosolic ribosomal hits were removed because they increased FRET in the Dead sensor, but only 4 of 25 (16.0%) of mitochondrial ribosomal subunits were removed for that reason. Of the 79 hits remaining in the glycolytic condition after removing artifacts, 43 (54.4%) that protected ATP in glycolysis also decreased ATP in the respiratory condition, indicating opposite effects of knockdown of the same genes in opposite metabolic contexts (Fig 3C).

Only 6 genes reduced the FRET signal of the ATP sensor in basal conditions, but none decreased FRET from the Dead sensor (S1, S2 Tables, S4 Fig). While 60 genes produced small increases in the ATP FRET signal in basal conditions, only 8 (13.3%) did not also increase FRET from the Dead sensor (S3 Table). The magnitude of phenotypes observed in the basal condition was smaller than seen in the respiratory or glycolytic conditions (Fig 3D, and S4 Fig). We conclude that very few genes alter ATP levels in basal condtions, consistent with the hypothesis that maintaining ATP levels is of primary importance in cells, and that cells adjust their metabolism and growth to maintain ATP levels. The acute metabolic perturbation in the respiratory and glycolytic conditions was thus essential to uncovering genes critical to maintaining ATP levels.

### Enrichment of mitochondrial transcription and translation genes among ATP-critical pathways

**Fig 4:**
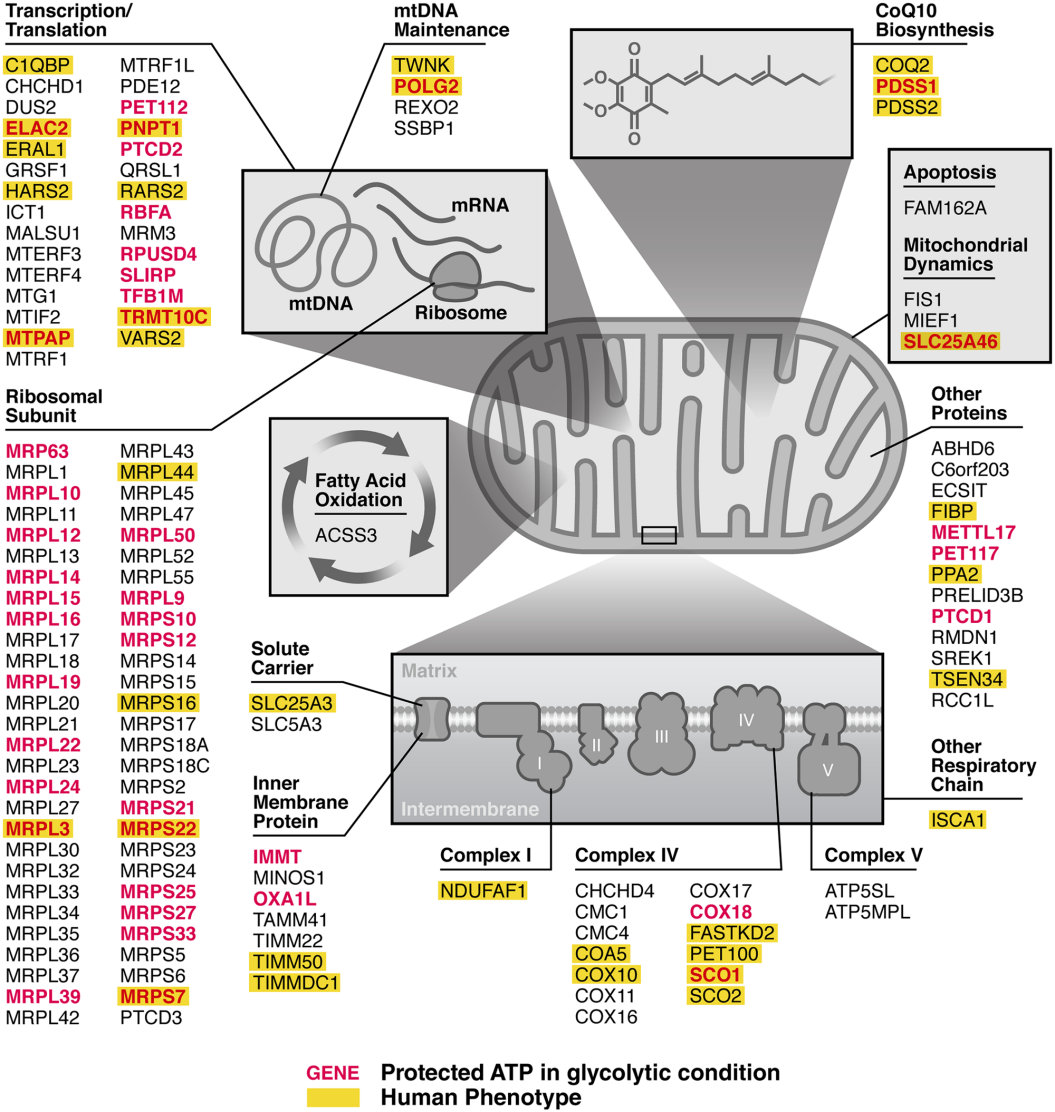
Mitochondrial pathways identified as critical to maintaining ATP. Genes identified as hits that decrease ATP when knocked down in the respiratory condition are grouped by their function within the mitochondria. Genes encoding components of the mitochondrial ribosomal subunits or other functions in transcription/translation were enriched among hits. Within the respiratory chain, genes encoding components of complex IV were enriched. Genes that, when knocked down, increased ATP in the glycolytic condition are in red lettering. Genes listed in OMIM (omim.org) as known to cause human mitochondrial disease are highlighted in yellow. Within each functional category, genes are arranged in alphabetical order. OMIM, Online Mendelian Inheritance in Man.

Our sublibrary contained genes from 3 cellular processes (vesicular trafficking, cell motility, and mitochondrial function) that behaved differently under respiratory conditions. As expected, few trafficking or motility genes affected ATP (Fig 3D), suggesting that many functions are not needed to maintain ATP. Genes in the nonmitochondrial pathways that did affect ATP (S3 Table) are potentially unrecognized mitochondria-related genes. Among genes involved in mitochondrial structure or function, specific pathways were clearly enriched (Figs 3D and 4). A total of 85% of protein subunits of mitochondrial ribosomes reduced ATP when knocked down. A similar pattern was seen with other genes involved in transcription and translation from the mitochondrial genome. A smaller fraction of respiratory chain genes impacted ATP when knocked down (23%, Figs 3D and 4) compared with the mitochondrial ribosome. Among these respiratory chain genes identified as hits, there was an enrichment for those involved in CoQ10 biosynthesis and copper insertion into complex IV (Fig 4 and S3 Table).

**Fig 5:**
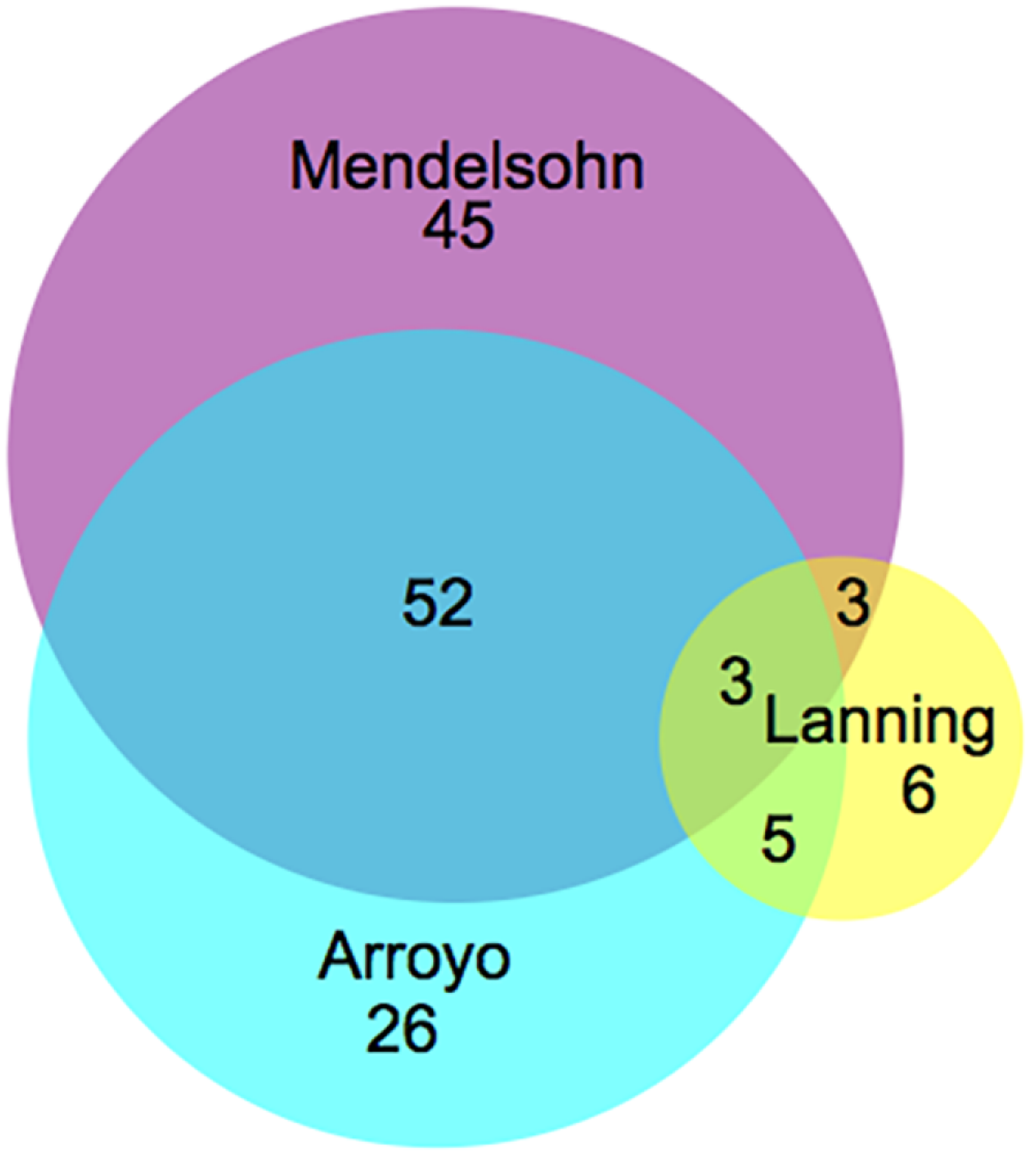
Overlap with reported gene knockdown screens on mitochondrial function. Venn diagram comparing low-ATP hits (Lanning and colleagues) or lethal hits (Arroyo and colleagues) from 2 related mitochondrial function screens [4,18] to ours in the respiratory condition. This analysis only included genes that were represented in all 3 screens. Hits examining the impact of glucose versus galactose substrate on cell death were derived from Arroyo and colleagues. S3 Table (FDR 0.3, the most inclusive cutoff). Hits examining the impact of 10 mM pyruvate on ATP levels by luciferase assay were derived from Lanning and colleagues S3 Table listing genes that altered ATP by >25%. FDR, false discovery rate.

In 2 previous studies, the effects of knocking down genes on ATP levels [4] and cell death as a function of metabolic substrate [18] were assessed by complementary methods. We compared our results to these screens and found overlap with our screen and the “death screen” conducted by Arroyo and colleagues, which examined genes necessary for cell survival in galactose, which forces reliance on mitochondrial metabolism (Fig 5). The agreement between our screen and that of Arroyo and colleagues suggests that many genes for ATP maintenance are also needed for cell survival in substrate that forces reliance on oxidative phosphorylation. While we found about two-thirds of the hits from the Arroyo screen, half of our hits were not found by Arroyo and colleagues, suggesting that some cells survive with impaired respiratory function and may not be identified by a survival or growth screen.

There was very little overlap with the study by Lanning and colleagues from either our screen or that of Arroyo and colleagues. We expected the pyruvate condition from Lanning and colleagues to overlap with our respiratory condition. Instead, even though our library and that of Lanning and colleagues screened 384 genes in common, there was an overlap of only 6 ATP-decreasing genes (Fig 5). Many ATP-decreasing genes in our study increased ATP in the study by Lanning and colleagues, overall suggesting that the assays produced different results.

### Low-ATP hits impair mitochondrial bioenergetics

**Fig 6:**
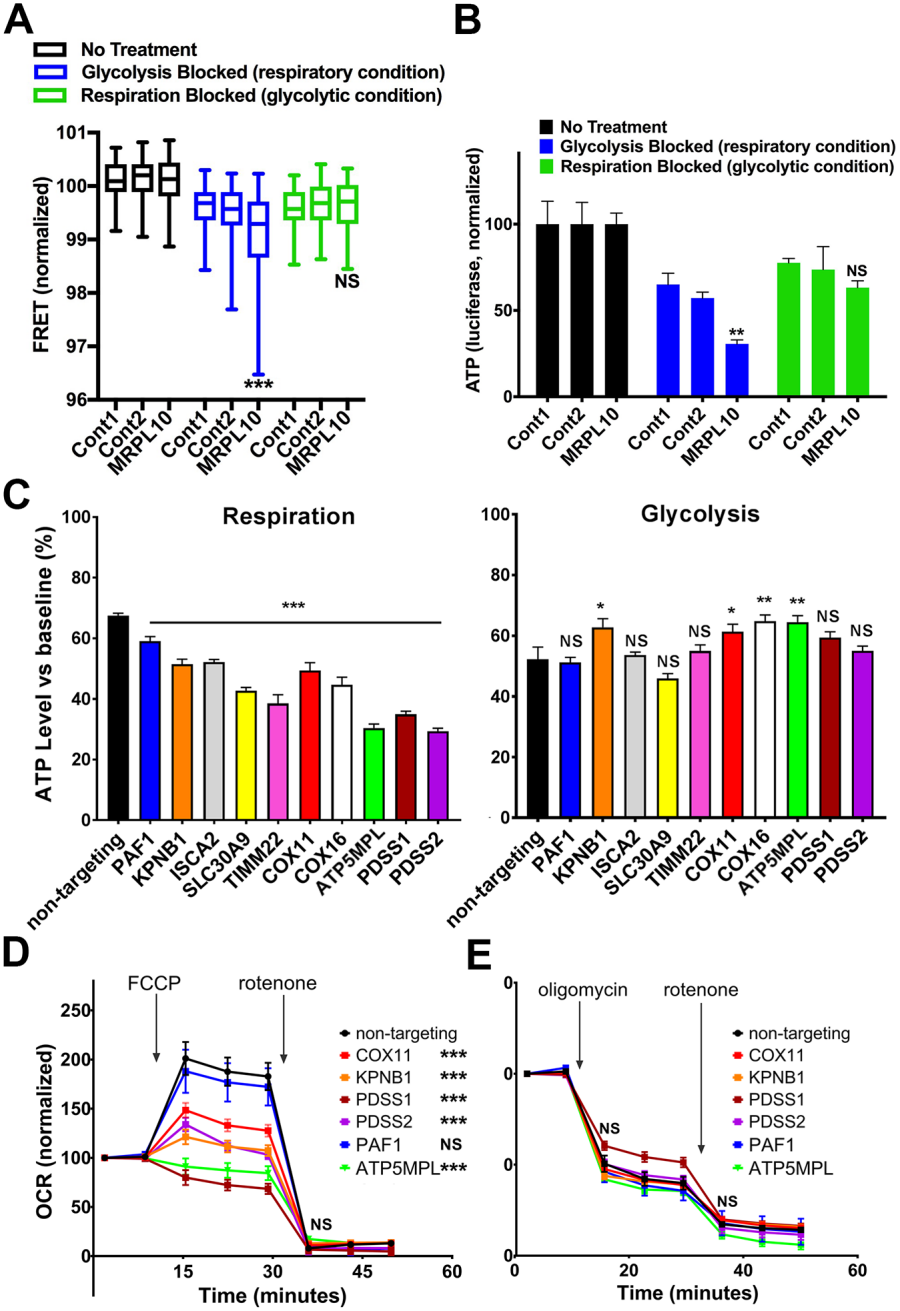
Validation and characterization of selected low-ATP hits in respiratory conditions. (A) K562 lines were generated expressing the dCas9-KRAB, Clover-mApple ATP FRET sensor, and 1 sgRNA against *MRPL10* or non-targeting control guides (Cont1, Cont2), and ATP levels were assessed by flow cytometry. Compared with the ATP level in basal conditions, *MRPL10* knockdown decreased ATP to a greater extent in respiratory conditions (box and whisker plots; line = median; box = 25th–75th percentile; whisker = 5th–95th percentile). In contrast, in glycolytic conditions, ATP levels dropped similarly in *MRPL10* and control groups. *N* = 3,128–11,971 cells examined per group for FACS. ***p < 0.001 versus both corresponding control groups by two-way ANOVA with Tukey multiple comparisons test. (B) Knockdown of *MRPL10* in K562 cells also decreased ATP levels by luciferase to a greater extent in respiratory conditions. In contrast, in glycolytic conditions, ATP levels again dropped similarly in *MRPL10* and control groups. Data show mean ± SEM; *N* = 4 wells per group from 2 independent experiments for luciferase. **p < 0.01 versus both corresponding control groups by one-way ANOVA with Dunnett multiple comparisons test. (C) K562 cells expressing dCas9-KRAB were stably transduced with a single sgRNA corresponding to the indicated gene, identified as reducing ATP in the primary FRET-based screen. Cells were selected for sgRNA transduction as in the primary screen, allowed to recover, and then acutely incubated in respiratory or glycolytic conditions as in the primary screen. ATP concentration was measured by luciferase (CellTiter Glo 2.0). Bars represent ATP levels in drug/substrate treatment (respiratory or glycolytic) relative to cells untreated for the same time period from the same pool of cells. *p ≤ 0.05; **p ≤ 0.01; ***p ≤ 0.001. Error bars are mean ± SEM; *N* = 10–32 wells/group, compiled from 2–4 experiments per cell line. (D, E) OCR (aerobic respiration rate) and ECAR (a surrogate of glycolysis) in K562 lines were measured using a 96-well Seahorse Extracellular Flux Analyzer. Arrows show the additions of the mitochondrial uncoupler FCCP (1 μM), the ATP synthase inhibitor oligomycin (1 μM), or the mitochondrial complex I inhibitor rotenone (1 μM). Knocking down of *COX11*, *KPNB1*, *PDSS1*, *PDSS2*, or *ATP5MPL* significantly decreased maximal respiration (after FCCP). Stars in legend box indicate significant difference of maximal respiration after FCCP (panel D). Oligomycin and rotenone additions similarly decreased OCR in all lines (panel E), indicating similar fractions of basal oxygen consumption devoted to mitochondrial respiration (oligomycin) and nonmitochondrial consumption (rotenone). Data are compiled from 4 experiments; *n* = 6–18 wells per group. Error bars represent SEM. NS, *p* > 0.05; *p < 0.005; **p < 0.01; ***p < 0.001; using one-way ANOVA with Dunnett multiple comparisons test. Further information about this figure can be found in S1 Data. Cont, control; dCas9, dead CRISPR-associated protein 9; ECAR, extracellular acidification rate; FACS, fluorescence-activated cell sorting; FCCP, carbonyl cyanide-*4*-(trifluoromethoxy)phenylhydrazone; FRET, fluorescence resonance energy transfer; KRAB, Kruppel-associated box; NS, not significant; OCR, oxygen consumption rate; sgRNA, single guide RNA.

To further validate our assay, we generated stable cell lines expressing single sgRNAs against selected genes identified as low-ATP hits under respiratory conditions, selected for transduced cells with puromycin, and further verified gene knockdown in a subset of lines by quantitative real-time reverse transcription PCR (qRT-PCR) (S5 Fig). As expected, knockdown of *MRPL10*, a strong low-ATP hit, decreased mitochondrial-derived ATP as detected by FACS/FRET (Fig 6A) and by luciferase versus a negative-control sgRNA (Fig 6B) but did not alter ATP in the glycolytic condition by either method (Fig 6A and 6B). Luciferase analysis of additional low-ATP hits from the primary screen also confirmed decreased ATP in the respiratory condition, while glycolysis-derived ATP was unchanged or protected (Fig 6C), confirming by a second method that the FRET-based screen identified true changes in ATP, both increased and decreased. The magnitude of ATP change detected by luciferase, however, was somewhat smaller overall than that detected by FRET, suggesting that the FRET screen is more sensitive than standard luciferase assays. We hypothesize that the increased sensitivity of the screen is due to the large number of cells analyzed individually compared with pooling cells in a lysate as is required by luciferase analysis.

We also used a Seahorse analyzer to determine whether an impairment in aerobic respiration underlies decreased mitochondrial-derived ATP. Consistent with this, several genes identified as decreasing ATP, particularly *KPNB1*, which has no known role in respiration, showed decreased maximal respiration (following carbonyl cyanide-*4*-(trifluoromethoxy)phenylhydrazone [FCCP]) (Fig 6D and 6E). *PAF1* knockdown did not significantly decrease maximal respiration (Fig 6D), suggesting either another role in ATP regulation for this gene or a milder effect on respiration below the sensitivity of this assay. Impaired respiration might result either from an intrinsic deficit in respiration or decreased mitochondrial content. However, all lines had similar mitochondrial mass (S6 Fig), as assessed by western blot on cell lysates against translocase of outer membrane 20 kDa subunit (Tom20) and NADH:ubiquinone oxidoreductase subunit S4 (NDUFS4), suggesting that mitochondrial-derived ATP levels are decreased due to an impairment in intrinsic mitochondrial function.

### Direct ATP screening provides insights into the energy requirements of cell growth

**Fig 7:**
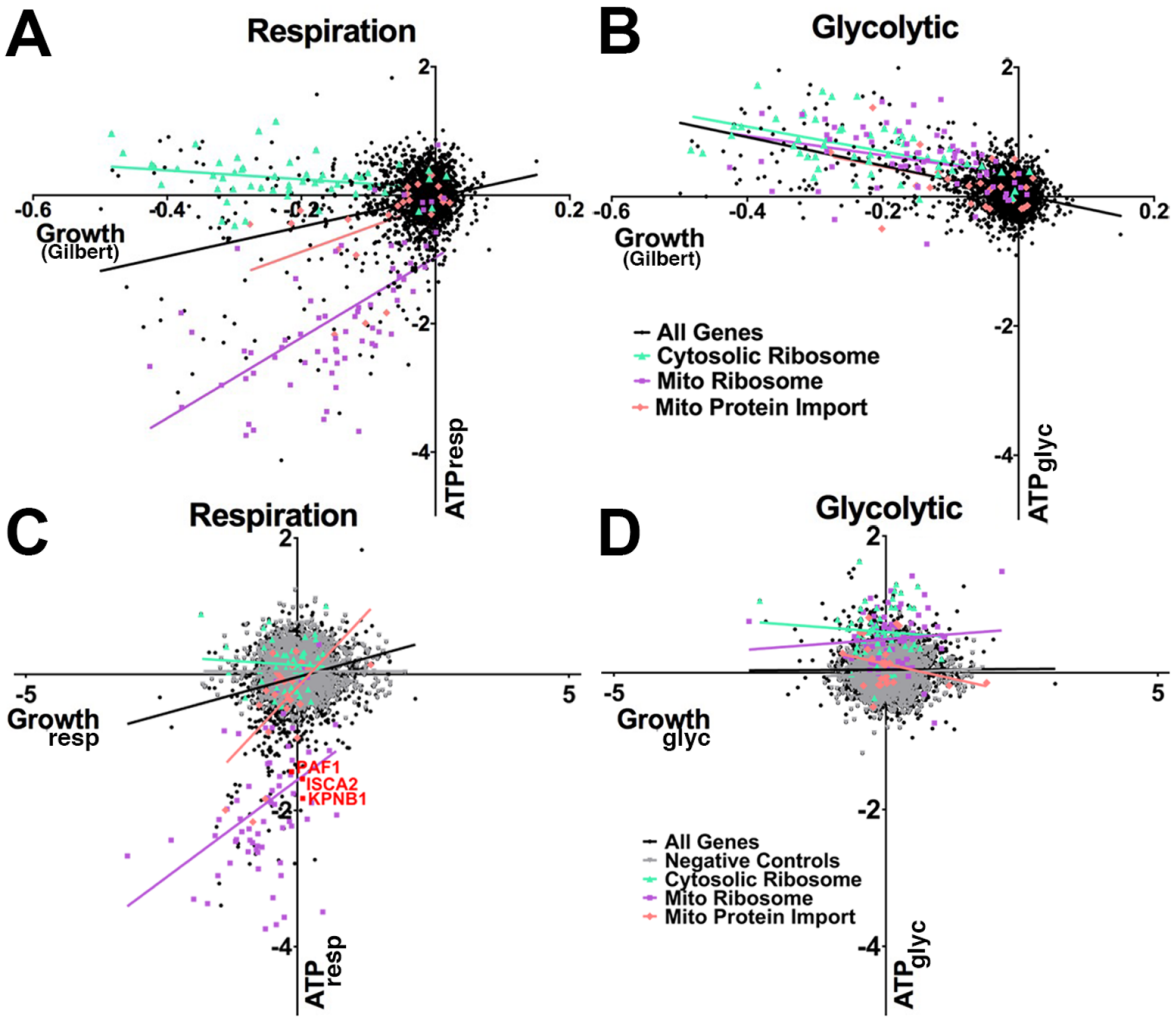
ATP from mitochondria promotes but is not always required for cell growth. (A, B) Genetic impact on growth can be separated as energy dependent and energy independent. ATP phenotypes in different metabolic contexts (y-axis, same data as in Fig 3) compared to growth phenotypes under basal conditions (x-axis, from Gilbert and colleagues 2014, S3 Table, column B) with the same CRISPRi library in the same K562 cell line. (A) When GROWTH_basGilbert_ was compared to ATP_resp_, genes encoding MRP subunits (purple squares) and MPI proteins (orange diamonds) had a strong positive correlation (6.26 ± 0.945 [*p* < 0.0001 versus null hypothesis] and 3.78 ± 0.163 [*p* = 0.0302], respectively) (i.e., among these gene knockdowns, decreasing ATP correlated with decreasing growth). Genes encoding cytosolic ribosomal subunits (CRP, green triangles) showed no correlation (−0.693 ± 0.371 [NS]). (B) When GROWTH_basGilbert_ was compared to ATP_glyc_, all 3 groups had negative slopes (MRP [−1.508 ± 0.503; *p* = 0.0039], MPI [−2.02 ± 0.962; *p* = 0.0474], and CRP genes [−1.91 ± 0.403; *p* < 0.0001]). (C, D) Genetic impact on growth is dependent on metabolic context. K562 cells expressing the CRISPRi library were grown in media favoring respiration or glycolysis, and the genes enriched and depleted in each metabolic condition were determined. (C) In respiratory conditions, when GROWTH_resp_ was compared to ATP_resp_, MRP (purple squares) and MPI (orange diamonds) genes had strong positive slopes (0.593 ± 0.164 [*p* = 0.0006] and 0.851 ± 0.252 [*p* = 0.0027], respectively, by F-test), and CRP genes (green triangles) did not (−0.0511 ± 0.1011; *p* = 0.6157). Several genes had low ATP but normal growth (red). (D) Under glycolytic conditions, when GROWTH_glyc_ was compared to ATP_glyc_, MRP, MPI, and CRP did not have significant slopes (0.060 ± 0.0833 [*p* = 0.471], −0.176 ± 0.135 [*p* = 0.587], and −0.061 ± 0.111 [*p* = 0.205], respectively). Further information about this figure can be found in S1 Data. ATP_gly_, ATP levels under glycolytic conditions; ATP_resp_, ATP levels under respiratory conditions; CRISPRi, clustered regularly interspaced short palindromic repeats interference; CRP, cytosolic ribosomal protein; GROWTHbasGilbert,; GROWTH_glyc_, growth under glycolytic conditions; GROWTH_resp_, growth under respiratory conditions; MPI, mitochondrial protein import; MRP, mitochondrial ribosomal protein; NS, not significant.

To determine what energy sources support cell growth, we analyzed our ATP phenotype data from different conditions (respiratory, glycolytic) compared with data from a published growth screen of the same CRISPRi library in K562 cells performed under basal conditions that permit both respiration and glycolysis [17]. When growth data are overlaid with our ATP data under respiratory conditions (ATP_resp_), genes organize into classes that differentially impact ATP and growth (Fig 7A). For example, knockdown of genes encoding components of the cytosolic ribosome slowed growth but did not affect ATP levels, but lower ATP levels did correlate with poorer growth for mitochondrial ribosomal genes (Fig 7A). In contrast to the respiratory condition, comparing growth with ATP levels under glycolytic conditions (ATP_gly_) (Fig 7B) demonstrated no relationship between growth and ATP levels for mitochondrial ribosomal genes. Therefore, among genes identified to impact growth, the ATP screen distinguishes genes that slow growth independent of energy levels from those that influence growth due to effects on mitochondrial function. Because cells in the growth screen had access to glucose without drugs (untreated), and we observed that few genes impact ATP levels in the similarly untreated basal condition (ATP_basal_) (S4 Fig), we conclude that mitochondrial function is important for K562 cell growth even when respiratory and glycolytic substrates are present and ATP levels are normal. In contrast to the respiratory condition, when growth was compared with the corresponding ATP levels under basal conditions (S7 Fig) or glycolytic conditions (Fig 7B), there was no relationship between growth and ATP levels.

To further understand how genes influence growth through energy-independent and -dependent mechanisms, we compared ATP and growth under matching metabolic conditions. We performed separate growth screens in respiratory (GROWTH_resp_), glycolytic (GROWTH_glyc_), and basal (GROWTH_basal_) conditions and compared the growth phenotypes to the ATP phenotype in the corresponding metabolic condition. Mirroring what we observed when comparing respiratory ATP with growth in a basal condition (Fig 7A and 7B), we observed that in respiratory conditions for both ATP and growth, knockdown of genes encoding components of the cytosolic ribosome showed no relationship between ATP and growth, indicating that these genes inhibit growth independent of energy level. In contrast, after knockdown of genes encoding components of the mitochondrial ribosome, the extent of decline in ATP_resp_ correlated with the decrease in GROWTH_resp_ (Fig 7C). In contrast, there was no relationship between ATP and growth in the glycolytic or basal conditions (Fig 7D and S7 Fig). These data suggest that knockdown of mitochondrial ribosomal genes impairs growth by affecting mitochondrial energy metabolism, either directly (ATP) or indirectly (redox status from respiratory chain function) rather than by other effects on mitochondria (e.g., Ca^2+^ buffering or reactive oxygen species [ROS] production), although these effects could also be regulated in a substrate-specific manner.

Gilbert and colleagues (2014) noted more genes that slowed growth than we did. The difference might be in the number of cell divisions. They included only a basal condition, which permitted many cell divisions. We found that cells grew poorly in respiratory and glycolytic conditions, limiting the total number of divisions. The ability to screen for phenotypes that are not compatible with robust cell growth is another advantage of FRET-based over growth-based screening.

Finally, although modest ATP decreases correlate with lower growth, several genes (e.g., *PAF1*, *KPNB1*, or *ISCA2*, Fig 7C) decrease ATP despite normal growth in respiratory conditions, again indicating that growth-inhibiting effects of decreased ATP can be overcome. Such genes that impair mitochondrial energy metabolism without slowing growth would be missed by growth-based screens but can be identified by a functional assay for ATP, as shown here.

### Genes identified in the ATP screen influence cancer cell proliferation

Modulating ATP levels may have therapeutic potential for diseases with high biosynthetic requirements, such as cancer. Yet little is known about how diverse cancers respond to metabolic therapies, and metabolic therapies are not yet major strategies employed in the clinic. By identifying energy-modulating genes in human tumors, our screening approach may help to identify those cancer-related mutations that are critical to cellular ATP levels and thus support cancer cell growth.

Based on the results of our growth screen, we reasoned that hits that disrupt mitochondrial-derived ATP would negatively impact cancer cell growth and examined 1 hit that decreased ATP in respiratory conditions named *METTL17*, which is implicated in breast cancer [19]. We cloned the sgRNA targeting sequence associated with the greatest ATP alterations for the *METTL17* (sequence provided in Materials and methods). Lentivirus expressing these or negative-control sgRNAs was transduced into HCC827 human lung cancer cells expressing dCas9-KRAB. After 1 week, total METTL17 protein levels were more reduced in cells transduced with sgRNA targeting this protein than in negative controls (S8A Fig).

Anticancer therapies inhibit tumor growth by affecting proliferation, and to determine whether *METTL17* silencing altered cell proliferation, we performed trypan blue staining of HCC827 cells expressing either negative-control sgRNA (Neg 1) or stable silencing of *METTL17* that were exposed to either control medium or medium containing 20 mM 2DG over 48 hours. *METTL17*-silenced cells yielded significantly fewer viable cells at basal condition than negative controls (S8B Fig), while the number of nonviable cells (trypan blue–positive) did not differ significantly between the groups. Inhibition of glycolysis with 2DG further decreased the total number of viable cells after 24 hours in both negative-control and *METTL17*-silenced lines (S8B Fig).

To determine whether reducing METTL17 protein expression compromises cell cycle progression either at baseline or during glycolysis inhibition, unsynchronized HCC827 cells in standard culture conditions were collected at 24 hours after exposure to control medium or medium with 10 or 20 mM 2DG. Collected cells were fixed, stained with propidium iodide, and analyzed by FACS. Baseline G1 and G2 fractions were similar between control and METLL17 cells at baseline (S8C Fig). Forcing control HCC827 cells to increase mitochondrial energy production by inhibiting glycolysis with 2DG led to an accumulation of cells in G1. This shift was absent in cells lacking METTL17 (S8C Fig), suggesting that knockdown of METTL17 decreases the overall growth rate, potentially to preserve ATP levels. We specifically assessed apoptosis by analyzing Annexin V using FACS, which quantified <6% Annexin V positivity across all cells and conditions, indicating that death was not prominent under basal or 2DG conditions (S8D Fig).

Although *METTL17* did not itself impact growth in K562 cells, it was nonetheless identified in our ATP screen performed in K562 human leukemia cells; these subsequent studies link *METTL17* to a cancer-relevant endpoint in a different cancer cell line—in this case, human lung cancer cells—in which stable silencing of *METTL17* significantly suppresses basal cancer cell proliferation without producing increased apoptosis. This finding parallels observations from our screen that indicate a negative relationship between genes that inhibit mitochondrial function and basal cell growth. From the standpoint of cancer therapeutics, these data raise the possibility of pharmacologic inhibition of METTL17 function or other genes critical to mitochondrial-derived ATP suppressing tumor growth, and future studies are needed to assess this possibility and its potential to preferentially target tumor cells.

### CoQ10 supplementation provides gene-specific protection against ATP depletion

Our screen might be used to identify specific genetic disorders that respond to targeted energy-based therapies. We focused on CoQ10, a lifesaving therapy for a subset of mitochondrial disorders caused by CoQ10 deficiency [5,20], widely used with many other mitochondrial disorders though with less consistent clinical benefit [6,21]. To assess the capacity of CoQ10 to restore ATP levels, we developed a mini-library of 180 sgRNAs consisting of 1 to 2 sgRNAs per gene, with the strongest ATP-lowering phenotypes in the primary respiratory screen, including 3 CoQ10-biosynthetic genes from the primary screen that lowered ATP in the respiratory condition (*PDSS1*, *PDSS2*, *COQ2*; CRISPRi against 7 other CoQ10 biosynthetic genes did not lower ATP in our main screen in respiratory conditions and were not included [see S1 Table for each gene phenotype]) as well as 20 non-targeting sgRNAs that had no effect on ATP level (see S4 Table for sequences of included sgRNAs). Cells were pre-incubated with 50 μM CoQ10 for 5 days and switched to respiratory conditions before sorting, as in the primary screen.

**Fig 8:**
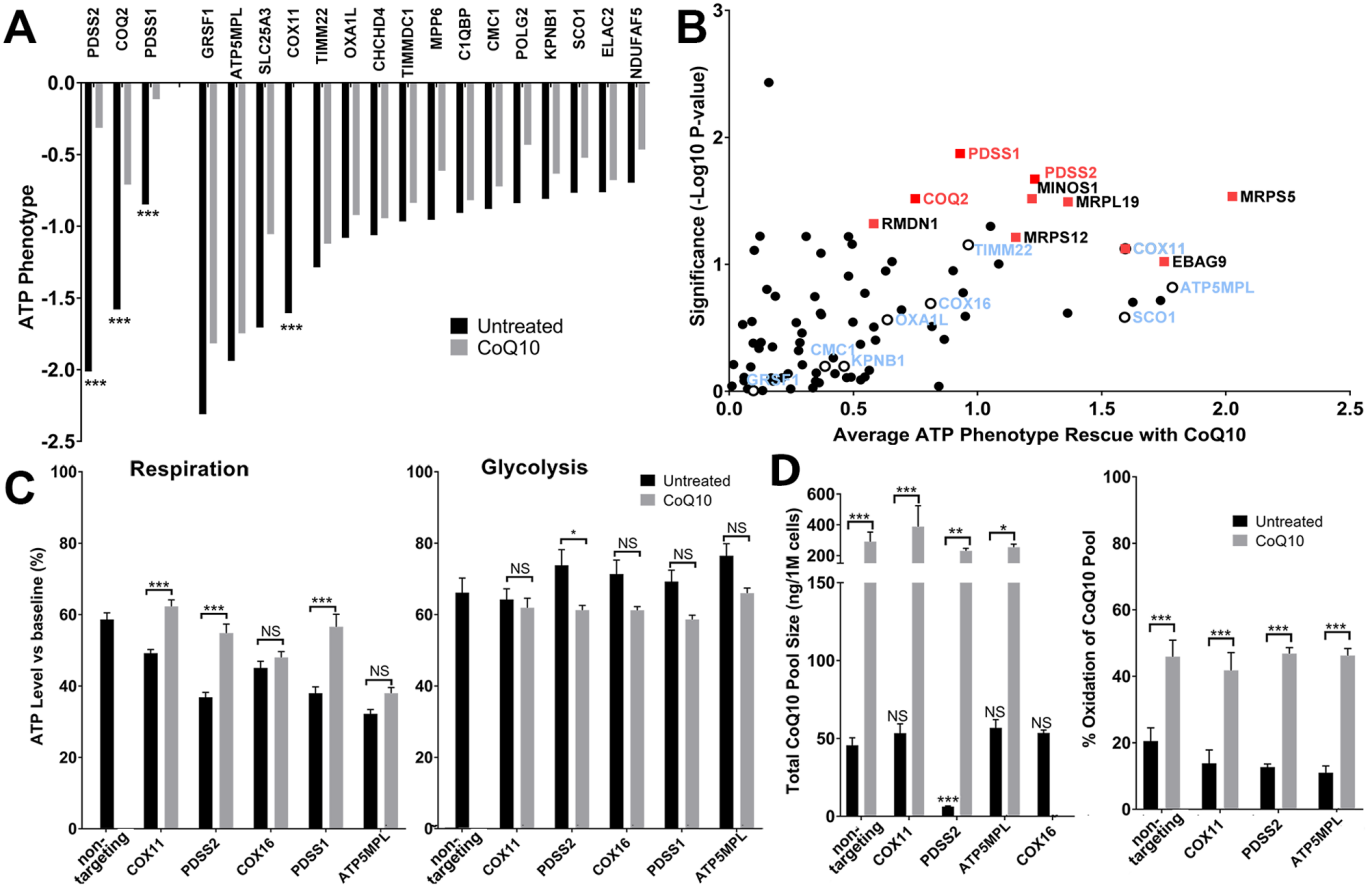
Identification of genes responsive to therapeutic CoQ10. (A) CoQ10 rescue of ATP in respiratory conditions was assessed with a mini-library of sgRNAs. Cells were pre-incubated with 50 μM CoQ10 for 5 days and then placed under respiratory conditions before sorting. Four genes showed statistically significant improvement in ATP levels (difference of untreated and CoQ10-treated ATP phenotype). Black bars indicate untreated ATP phenotypes, and gray bars show the ATP phenotype with CoQ10. *PDSS2*, *COQ2*, and *PDSS1* have biosynthetic roles in CoQ10, and CoQ10 blocks the decrease in ATP from knocking down these genes. CoQ10 also significantly blocked the low-ATP phenotype of *COX11*, which has no known CoQ10 biosynthetic role. Data from 2 experiments, 1–2 guides studied per gene, 1 million cells sorted per group. ***p < 0.001 versus non-targeting guides (with a phenotype of 0 and no change in response to CoQ10) by one-way ANOVA with Dunnett multiple comparisons test. (B) Plot of genes identified as decreasing ATP in the initial screen for rescue with CoQ10 supplementation. Genes that decreased ATP in the respiratory screen were reanalyzed with and without supplementation with CoQ10. The positive (rescue) portion of the graph is shown. x-Axis is fold-rescue by CoQ10 (phenotype in CoQ10 minus phenotype in vehicle). y-Axis is the -log_10_ of the *p*-value (*t* test on null hypothesis that the rescue was 0) based on the multiple sgRNAs tested for that gene. The figure consists of 3 repetitions of the subgenome library screen with CoQ10 and 2 without. Genes showing strong rescue with CoQ10 (either having a *t* test *p*-value of <0.05 and rescue larger than 1 SD from the average rescue of non-targeting guides, or a *t* test *p*-value of less than 0.1 and a rescue larger than 2 SDs from the average rescue of non-targeting guides) are labeled with red squares. CoQ10 biosynthetic genes are labelled with red text. Other genes that were investigated in other parts of this figure are labelled with hollow circles and blue text. (C) Rescue of low ATP by CoQ10 as measured by luciferase. K562 cells expressing dCas9-KRAB and a single sgRNA were placed in respiratory or glycolytic conditions as in the primary screen. ATP concentrations were measured by luciferase as in Fig 6. Data show mean ± SEM; *N* = 32 wells per group compiled from 4 independent experiments. *p < 0.05; ***p < 0.001 between untreated and CoQ10-treated conditions by two-way ANOVA with Dunnett multiple comparisons test. (D) K562 cells expressing dCas9-KRAB and a single sgRNA were collected, and total CoQ10 and CoQ10H2 levels were determined by HPLC and mass spectrometry with and without supplementation of 50 μM exogenous CoQ10 in the cell culture medium. The same cells assayed for total CoQ10 were also examined for the fraction of total CoQ10 in the oxidized state with and without CoQ10 supplementation. Untreated CoQ10 total pool sizes were compared with the untreated non-targeting guide by one-way ANOVA with Dunnett multiple comparison correction. The effect of CoQ10 treatment on total pool size and CoQ10 pool oxidation was analyzed within each gene knockdown line by two-way ANOVA with Dunnett multiple comparison correction. Data show mean ± SEM; *N* = 7–9 replicates per group compiled from 3 independent experiments. *p < 0.05; **p < 0.01; ***p < 0.001. Further information about this figure can be found in S1 Data. CoQ10, coenzyme Q10; dCas9, dead CRISPR-associated protein 9; HPLC, high-performance liquid chromatography; KRAB, Kruppel-associated box; NS, not significant; sgRNA, single guide RNA.

CoQ10 supplementation protected ATP levels in all 3 CoQ10 biosynthesis genes identified as reducing ATP in the primary screen in the respiratory condition (*PDSS1*, *PDSS2*, *COQ2*) (Fig 8A), demonstrating the specificity of the assay to mitochondrial function and that our assay is able to detect interventions that protect ATP. CoQ10 had no effect on the decrease in ATP produced by CRISPRi against most genes not in the CoQ10 pathway. One notable exception, *COX11* (a component of cytochrome c oxidase [22] not known to be involved in CoQ10 biosynthesis), responded to CoQ10 supplementation, suggesting that CoQ10 rescues bioenergetic function by some mitochondrial genes not known to be involved in CoQ10 biosynthesis.

We then repeated the full subgenome library screen 3 times with CoQ10 supplementation and twice without. Known CoQ10 biosynthetic genes again showed improved ATP with CoQ10 supplementation, and a subset of low-ATP hits with no known direct function in CoQ10 biosynthesis including *COX11* showed improved ATP with CoQ10 supplementation (Fig 8B, S5 Table). To support these findings, we measured ATP levels with and without CoQ10 in a subset of these genes by luciferase and confirmed rescue of ATP levels in several genes (*PDSS1*, *PDSS2*, *COX11*) and no rescue in others (*COX16*, *ATP5MPL*) (Fig 8C), consistent with what was observed by FRET (Fig 8A and 8B). No improvement in ATP was detected with CoQ10 supplementation in the glycolytic condition by luciferase (Fig 8C), confirming the specificity of CoQ10 to the respiratory metabolic context.

CoQ10 could rescue ATP levels by correcting a primary or secondary CoQ10 deficiency or by augmenting mitochondrial function through other mechanisms. To distinguish between these possibilities, we measured cellular CoQ10 levels with and without CoQ10 supplementation in cells with a CoQ10 biosynthetic gene knocked down (*PDSS2*) or selected low-ATP hits with no known function in CoQ10 biosynthesis (*COX11*, *ATP5MPL*, *COX16*). We observed that baseline CoQ10 levels were low in cells lacking *PDSS2*, but CoQ10 levels in cells lacking *COX11*, *ATP5MPL*, or *COX16* were not different from control cells (Fig 8D). Supplementation with CoQ10 increased CoQ10 levels in all cells (Fig 8D). Supplementation also increased the fraction of total CoQ10 in the oxidized versus reduced state in control cells and in cells with single-gene knockdown (Fig 8D). From these data, we conclude that CoQ10 supplementation can improve mitochondrial ATP production by correcting a primary CoQ10 deficiency, but the rescue of *COX11*-induced ATP deficiency results either from additional improvements in respiration or other undefined benefits of supraphysiologic CoQ10 levels.

## Discussion

### Specificity of ATP FRET screen

Our screen identified genetic factors that regulate ATP with high specificity. None of the >2,000 simulated genes we screened produced hits, and 87.2% (136/156) of our total hits that decreased ATP under respiratory conditions had known mitochondrial functions. The other 12.8% (20 genes) likely also function in energy metabolism and warrant further study. CRISPR and RNAi screens using whole-genome libraries are common and powerful tools to dissect genetic pathways in mammalian cells. Yet most assay an endpoint (e.g., survival, growth, reporter gene expression) that may reflect heterogeneous molecular intermediates. Measuring a metabolite in real time at a scale compatible with genome-wide screening is novel and enables a mechanistic dissection of detailed biochemical processes.

Genes identified in our screen had little overlap with the only screen published on genetic regulators of ATP (Lanning and colleagues) [4]. This study did not observe decreased ATP phenotypes under a respiration-only condition (pyruvate) with inhibition of most mitochondrial ribosomal genes, and in fact, 6 such genes increased ATP by more than 25% when knocked down, and they found increased ATP with inhibition of a majority of screened CoQ10 biosynthetic genes. While some differences may be attributable to the different cell types used (K562 cells in this study; HeLa in Lanning and colleagues’ study), the mitochondrial ribosome and CoQ10 biosynthesis are believed to be critical to aerobic respiration across different cell types [20,23]. The discrepancies between these studies warrant further consideration. Lanning and colleagues measured ATP in many cells concurrently, and ATP was normalized to total DNA content. This method would be susceptible to artifacts caused by differences in cell size (altering the absolute ATP content) or cell cycle (impacting the ATP/DNA ratio) that would not reflect changes in ATP concentration. Other differences include the use of 2 siRNAs per gene by Lanning and colleagues versus 10 sgRNAs per gene in this study, and the use of an ATP-insensitive control to remove ATP-independent artifacts in this study. We believe that the methodologic advantages detailed here—including our detection of nearly all genes in one mitochondrial compartment (i.e., the ribosome) and the gene-specific rescue of ATP by CoQ10—demonstrate that our FRET-based method of screening for ATP modifiers adds substantially to the nascent field of metabolite-based screening.

The high-throughput screen described here required fast-growing cells capable of effective CRISPRi-mediated gene knockdown. Any cell type selection, particularly a transformed cell line, will likely impact the results of a screen. K562 cells have successfully revealed important biology in other related screens [17,18], and here, they demonstrated clear pathway-specific effects, e.g., from knockdown of mitochondrial ribosomal subunits. These effects are biologically plausible, are supported by additional investigation (e.g., rescue by CoQ10), and likely can be generalized to other cell types. Knowledge of essential and novel mitochondrial processes enables future detailed studies in targeted disease models.

Effects of pH on the fluorescent ATP biosensor are unlikely to underlie the findings reported here. We employed a Dead sensor control to eliminate artifacts due to ATP-independent effects on the fluorophores, and many hits identified in this study were validated by luciferase assay, a completely orthogonal methodology.

### ATP levels are uniquely sensitive to disruption of mitochondrial ribosomal proteins

We found differences in the classes of genes required to maintain ATP levels. Mitochondrial-derived ATP levels were decreased by knockdown of 40% (27/67) of mitochondrial transcription/translation genes and 85% (57/67) of mitochondrial ribosomal genes. In contrast, knockdown of only 23% (21/90) of screened respiratory chain genes decreased ATP levels. The specific respiratory chain genes identified in this screen may be critical to maintaining ATP levels. Indeed, mutations in some of the genes identified in our screen, e.g., *PDSS2* and *COX10*, are believed to cause human mitochondrial disorders by depleting ATP [24,25].

Our screen also assayed other classes of mitochondrial genes with known roles in promoting respiration, but only a subset of the genes tested were identified as hits. For example, our screen identified select components of the mitochondrial contact site and cristae organizing system (MICOS) complex, which plays a critical role in maintaining cristae morphology (*IMMT* [26], *MINOS1* [27]), and a subset of mitochondrial fusion and fission proteins (e.g., neither Mfn1 or Mff were low-ATP hits). In one case, the fission receptor encoded by *MIEF1* (Mid51) was a hit, while the related gene *MIEF2* (Mid49) was not, despite functioning in the same pathway. There are likely several reasons why certain genes with known functions in respiration were not hits. First, in many cases, these genes are likely less critical for maintaining ATP levels than those genes identified as hits. For example, although mitochondrial dynamics proteins influence respiration, mitochondria can continue to respire with different morphologies [28], and the impact on respiration may only be observed in cell types and conditions with high energy requirements [29]. In other cases, there may be compensatory pathways that maintain respiration or delayed effects on ATP levels that would be missed in our assay system. We examined the capacity to maintain ATP after only a few days of gene knockdown and could have missed genes that might influence ATP levels more slowly, e.g., by compromising processes such as mitochondrial DNA maintenance [30]. In other cases, there may have been insufficient knockdown by CRISPRi, or gene function may be preserved even with substantial knockdown. As such, future studies will be needed to determine whether specific pathways (e.g., individual respiratory chain complexes) are more or less critical for ATP synthesis. Our finding that a majority of mitochondrial ribosomal subunits were identified as hits suggests that most genes critical for ATP maintenance would be identified in this screen and that many genes not hit may indeed be less critical for maintaining ATP in the conditions screened in this study.

Why are ATP levels so sensitive to disruption of genes involved in mitochondrial protein synthesis? Genes in the mitochondrial genome are retained evolutionarily, including proteins at the core of respiratory chain complexes [31], whereas peripheral subunits were more easily moved to the nucleus, perhaps due to differences in protein hydrophobicity. Disrupting nuclear-encoded genes that impair mitochondrial transcription and translation may affect mitochondria more than individual respiratory chain components because they interfere with expression of multiple core, mitochondrially encoded subunits. This observation suggests that combined respiratory defects cause disease due to more severe energy failure than dysfunction of a single respiratory chain complex.

Mutations in only a few mitochondrial ribosomal proteins cause human disease [23]. Others have likely not yet been linked to disease due to rarity or early lethal phenotypes [32]. Indeed, whole-exome sequencing has led to the discovery of mutations in mitochondrial ribosomal subunits as the cause of neonatal lethal diseases [33]. From our findings, loss of these proteins may produce disease due to energy failure, but their loss could also compromise other mitochondrial functions, including Ca^2+^ metabolism and ROS production, and deficits in these functions may contribute to disease.

The screen presented here was not exhaustive of all mitochondria-localized genes (see S1 Table for complete gene list). For example, most components of the tricarboxylic acid (TCA) cycle and some respiratory chain subunits, including many complex III subunits, were not included, and further investigation is required to define the bioenergetic requirement for these genes and for comparisons of individual respiratory chain complexes.

### New genes linked to metabolism

We identified genes without recognized mitochondrial functions that, when knocked down, reduced ATP only from mitochondria. The strongest such hit was *KPNB1*, which encodes a component of the nuclear pore complex. No human disease is yet associated with this gene, but it may be needed for mitochondrial biosynthesis [34] because *Drosophila* larvae lacking Importin-Beta had fewer mitochondria. This may be due to impaired nuclear import of Nrf2 [35]. Our studies show impaired mitochondrial function with *KPNB1* knockdown (Fig 6C and 6D), indicating a conserved role in mitochondrial metabolism but normal mitochondrial mass (S6 Fig), suggesting that the defect is not due to decreased mitochondrial biosynthesis. Also, *PAF1*, which regulates nuclear gene transcription [36], has no known link to energy metabolism, but its knockdown also decreases ATP only when ATP is produced by the mitochondria as detected by FRET and luciferase (S1, S3 Tables, S3 Fig, Fig 6C). Overall, our findings demonstrate the capacity of functional screens to identify mitochondria-related genes whose protein products may not necessarily be localized to the mitochondria as well as demonstrate that further study of putative mitochondrial genes may give insight into how energy levels are maintained, contribute to disease, and/or can be therapeutic targets.

### Metabolic substrate determines gene requirements for ATP levels

Knocking down most mitochondrial ribosomal protein genes affected mitochondria-derived ATP levels but did not decrease ATP when cells used glycolysis alone or both respiration and glycolysis. Therefore, glycolysis may compensate for decreases in aerobic respiration, and/or energy consumption may be decreased to maintain normal ATP. In support of the former, when cells were forced to use only glycolysis-derived ATP, knockdown of some mitochondrial ribosomal proteins actually enhanced the capacity of cells to maintain ATP levels above those of control cells in the same condition (Fig 3C and 3D). This likely reflects up-regulated glycolysis when aerobic respiration was compromised by gene knockdown.

Which metabolic context is most relevant to in vivo human diseases of energy failure? This is poorly understood. Local substrate conditions are often not known and differ among cell types, energy requirements, stressors (e.g., starvation), and perhaps genetic modifiers of metabolism. Developmental stage is a consideration; e.g., the in utero environment is more hypoxic than after birth, leading to increased reliance on glycolysis during development [37].

In our experiments, knockdown of only a few genes caused chronic basal ATP deficiency. More were important to maintaining ATP levels during acute metabolic perturbations. We hypothesize that disease more often results from acute insults to ATP homeostasis in a setting that is sensitized by an underlying deficiency in mitochondrial function than a chronic deficit in ATP levels. Acute exposure to respiratory or glycolytic conditions may not seem representative of chronic metabolic disease, but many disorders of energy failure manifest as intermittent acute clinical episodes rather than chronic disease. For example, patients with Leigh syndrome or mitochondrial encephalopathy, lactic acidosis, and stroke-like episodes (MELAS) present with acute stroke-like episodes [38]. Similarly, hypoxia/ischemia, rhabdomyolysis due to mitochondrial disease, and hypoglycemia due to inborn errors in fatty acid oxidation are acute forms of energy failure and injury [39,40]. The relative contributions of chronic versus intermittent ATP perturbation to metabolic disease need more study.

### ATP correlates with, but is not required for, cell growth

What are the cellular consequences of insufficient ATP? ATP is required for many cell functions (e.g., neuronal activity, myocardial contractility, insulin release, and cell growth) [41]. However, it is rarely proven that insufficient ATP itself underlies deficits in these functions. Growth-based screens in human cells with shRNA or CRISPR libraries [17,42,43] create a “black box” between the gene and growth change that may not reflect a metabolic etiology. To investigate this, we studied the relationship of ATP level and growth under different metabolic conditions. Our data suggest that specifically insufficient ATP (i.e., energy failure), not loss of other functions, explains why decreasing mitochondrial ribosomal proteins compromise growth in respiratory conditions. Moreover, cells may decrease growth as a means of preserving ATP levels when ATP production is decreased. The relationship between ATP level and cell growth also raises the possibility of an energy threshold for growth. In addition, we identified several genes for which CRISPRi markedly decreased ATP without compromising growth, or slowed growth without affecting ATP. These findings highlight the significant utility of the ATP screen in beginning to define the contribution of metabolism to cellular functions.

An important strength of our screen is the ability to identify ATP-modulating genes under distinct metabolic conditions (basal, respiratory, glycolytic). Moreover, we hypothesize that genes identified in these specific contexts (metabolic and/or cellular) will exert similar effects in other cellular and experimental contexts. For example, our data indicate that *METTL17* loss significantly suppresses human lung cancer cell growth and support the idea that the identification of energy-modulating genes could inform the development of cancer-relevant biochemical approaches. Moving forward, defining the precise cellular and metabolic contexts amenable to metabolic-based therapy will require cancer-type–specific studies, as our findings suggest.

### CoQ10 responsiveness

To our knowledge, we report here the first high-throughput screen of responsiveness to CoQ10 (or any drug) for single-gene causes of mitochondrial dysfunction. Of at least 18 known CoQ10 biosynthetic genes [44], 10 were screened in our library, and 5 are known to cause human disease. Among these 5, knockdown of *PDSS1*, *PDSS2*, and *COQ2* decreased ATP specifically in the respiratory condition in this screen. CoQ10 supplementation prevented the drop in ATP in respiratory conditions in all 3 of these genes as detected by repeat FRET-based screening (Fig 8A and 8B) and by luciferase (Fig 8C). However, many mitochondrial disorders have not responded clinically to CoQ10 supplementation [6]. We also identified several genes in which mitochondrial-derived ATP increased with CoQ10, even in the absence of a known role in CoQ10 biosynthesis. In particular, *COX11* consistently showed improved ATP with CoQ10 supplementation (Fig 8A–8C). Moreover, cells lacking *COX11* did not have lower CoQ10 content, suggesting that the ATP rescue must be due to a role of CoQ10 in other mitochondrial functions. We speculate that supraphysiologic levels of CoQ10 may improve mitochondrial ATP production, e.g., by improving the efficiency of the respiratory chain [45] or increasing mitochondrial biogenesis [46]. Indeed, CoQ10 has been reported to partially prevent ATP depletion from mitochondrial stressors in the absence of a known defect in CoQ10 biosynthesis [47]. Alternatively, CoQ10 also has known functions as an antioxidant that could decrease energy requirements or indirectly increase energy production. Although *COX11* is not yet associated with disease, we hypothesize that *COX11* deficiency, if found, may respond to CoQ10 treatment.

If supraphysiologic CoQ10 can boost mitochondrially derived ATP, why is CoQ10 not more broadly effective? Perhaps there are 2 CoQ10 thresholds to increase ATP: one to restore deficient levels in disorders of CoQ10 biosynthesis and a second to boost mitochondrial ATP production though other mechanisms at supraphysiologic levels. Difficulty achieving high enough CoQ10 levels may explain the lack of clinical efficacy in humans, in which the bioavailability of CoQ10 is limited, especially in certain tissues, such as the brain [48]. Secondary CoQ10 deficiency may also underlie some mitochondrial disorders [49], contributing to the degree of CoQ10 responsiveness.

Our findings here suggest that the beneficial effects of CoQ10 supplementation on mitochondrial-derived ATP levels may also go beyond simply restoring CoQ10 levels to normal. Moreover, our observation that CoQ10 rescues ATP levels caused by knockdown of some genes and not others has important implications for the utility of high-throughput screening for targeting therapies to mitochondrial disease and other disorders of energy failure. There are hundreds of single-gene mitochondrial disorders, and until now, no mechanism existed to screen for effective interventions for each genetic deficiency individually. The high-throughput nature of our technology should permit preclinical screening of any number of candidate therapies in different substrate/drug combinations, adding tremendously to our understanding of the interaction between single-gene mitochondrial dysfunction and external stresses and therapies. Furthermore, just as we combined our ATP data with other large-scale growth screens to achieve new insights, metabolite screening for other molecules with fluorescent biosensors (e.g., ROS, nicotinamide adenine dinucleotide) may advance a genome-scale understanding of the regulation of metabolism.

## Materials and methods

### Cell lines

The ATP screen was conducted using K562 cells provided by Jonathan Weissman’s lab, identical to those used by Gilbert and colleagues 2014, with stable integration of dCas9-KRAB as described (ATCC 536 [RRID:CVCL_0004], a female cell line) [17,50]. K562 cells were maintained at 37 °C in RPMI-1640 with 25 mM HEPES, 2.0 g/L NaHCO_3_, 0.3 g/L L-glutamine supplemented with 10% fetal bovine serum (FBS), 2 mM glutamine, 100 units/mL penicillin, and 100 mg/mL streptomycin. Lentivirus expressing the Clover-mApple ATP or Dead sensors was produced by the UCSF Viracore and transduced with polybrene (8 μg/mL) into K562-dCas9-expressing cells via spinfection. Two days after transduction, cells were selected by FACS that expressed both fluorophores (Clover and mApple). To avoid artifacts from potential mutations or variations in a single cell, a small polyclonal population of ATP FRET sensor-expressing cells was expanded for all subsequent experiments. The use of a polyclonal population was very unlikely to have affected screen results for several reasons, as follows: post-FACS comparisons showed no difference in FRET distribution from small or large collections of ATP FRET sensor-expressing cells; sufficient cells were screened such that each sgRNA was screened in approximately 200 cells, which would average out any cell-to-cell variability; and, as described above, the Clover-mApple sensor did not differ in FRET signal based on sensor expression level.

For the CRISPRi pooled library, lentivirus was transduced as above, and 2 days after transduction, puromycin selection (0.65 μg/mL) was started and maintained for 4 to 5 days total, followed by 1 to 3 days of recovery from puromycin. Experiments were performed using the pooled sgRNA-expressing cells surviving antibiotic selection.

Cell lysates used for in vitro FRET experiments were obtained from COS7 cells originally obtained from Robert Edwards (UCSF) (RRID:CVCL_0224). COS7 cells were grown at 37 °C in high-glucose DMEM supplemented with 110 μg/mL penicillin and streptomycin and 10% FBS.

The human lung adenocarcinoma HCC827 cell line was originally obtained from Trever Bivonas (UCSF) (ATCC 2868, 39-year-old Caucasian female individual). HCC827 cells were grown at 37 °C in RPMI medium with 10% FBS, 1% penicillin/streptomycin, 1.5 mM pyruvate, and 0.05 mg/mL uridine. Lentivirus expressing dCas9-KRAB and sgRNA of interest was produced by the UCSF Viracore and transduced with polybrene (8 μg/mL) into HCC827-dCas9–expressing cells over 2 consecutive days. At 48 hours after transduction, puromycin selection (1 μg/mL) was started and maintained for 7 days total. Experiments were performed using the pooled cells surviving antibiotic selection. The cells were authenticated based on the knockdown of gene expression.

### Molecular biology

CFP-Venus ATP (AT1.03^YEMK^) and Dead (AT^R122K/R126K^) FRET sensors (which use a variant of CFP [mseCFP] as the donor fluorophore and YFP [circularly permuted mVenus] as the acceptor fluorophore, surrounding an ATP-binding protein) were kind gifts from Hiromi Imamura (Kyoto University) and Hiroyuki Noji (Osaka University) [7].

Clover-mApple ATP and Dead FRET sensors were constructed using Clover (Addgene number 40259), which was circularly permuted by PCR to begin at residue 173 with a linker (GGSGG) as described [51]. To the 3′ of a PCR product encoding the permuted Clover was ligated the ATP or Dead sensor domains, followed by mApple. The entire 5′-Clover-sensor-mApple-3′ construct was then subcloned into the lentiviral vector FUW2.

### Calibration of FRET sensors with cell lysates

COS7 cells were electroporated with DNA encoding the ATP FRET sensor, Dead sensor, or the individual fluorophores using a GenePulser (Bio-Rad, Hercules, CA) set for mammalian cells, 250 V, 950 μF, and allowed to grow for 2 to 3 days after transfection. Cells were then collected by scraping the plates and sonicating cells in SH buffer (10 mM HEPES, 0.32 M sucrose [pH 7.4]) and pelleting the remaining debris. Lysates were saved at −80 °C until use and were not refrozen. Thawed lysates were distributed in a 96-well plate, and fixed concentrations of MgATP (A9187, Sigma, St. Louis, MO) were added, all diluted in SH buffer. The pHs of the SH buffer and MgATP stock were adjusted with NaOH or HCl. Fluorescence was detected on a Molecular Devices SpectraMax M5 plate reader using wavelengths (ex/em) 550/610 (acceptor), 490/550 (donor), and 490/600 (FRET). Bleed-through into the FRET channel was subtracted by measuring single fluorophores [52].

### ATP FACS screen and analysis

#### Flow cytometry

To acutely force reliance on respiration and/or glycolysis prior to flow cytometry or luciferase experiments, cells were resuspended in PBS as follows: (1) respiratory condition: 2% FBS, 10 mM pyruvate plus 10 mM 2DG (Sigma, catalogue number D6134); (2) glycolytic condition: 2% FBS, 2 mM glucose plus 5 μM oligomycin plus 3 mM 2-deoxyglycose; or (3) basal condition: 2% FBS, 10 mM glucose plus 5 mM pyruvate with no drugs. All flow cytometry experiments and screening were conducted on a BD FACSAria II. The donor fluorophore (Clover) was excited using a 488-nm laser and detected using a 525/50-nm filter. FRET was detected by excitation from the 488-nm laser and emission via a 610/20- or 615/30-nm filter. For the CFP-Venus ATP sensor, excitation was from a 405-nm laser and FRET detected by a 525/50-nm filter. mTagBFP fluorescence from the CRISPRi library was excited by a 405-nm laser and detected by a 450/50-nm filter. Donor and FRET channels were detected on a linear scale, and mTagBFP fluorescence was detected on a logarithmic scale. Sorting was conducted using 4-way purity into 2 tubes and an 85-μm nozzle. At least 200 cells/sgRNA in the sublibrary were collected in each repetition.

#### Gating paradigm

First cells were gated by forward (FSC-A) and side scatter (SSC-A), then for single cells using FSC-A/FSC-W and SSC-A/SSC-W. Next, cells were gated based on positive mTagBFP fluorescence, indicating the presence of a CRISPRi sgRNA, followed by gating out of cells with donor or FRET signal from the ATP sensor that overlapped with background (too dim) or exceeded the range of the sorter (too bright). The ratio of FRET/Donor was displayed as a histogram, and the top and bottom 25% of cells on this histogram were separated by FACS and collected for sequencing.

#### DNA preparation and sequencing

Cells collected by FACS were centrifuged, and pellets were frozen at −20 °C until processing. Genomic DNA was isolated using the Macherey-Nagel NucleoBond Xtra Midi Plus (Macherey-Nagel, Germany). The sgRNAs were amplified, and adaptors were attached in a single PCR step. The amount of 1.1 μg of undigested genomic DNA was used per 50 μL PCR reaction, and sufficient reactions were performed to include all isolated genomic DNA. PCR was conducted using Q5 HotStart High Fidelity Polymerase (NEB, Ipswich, MA) using the following forward primer: aatgatacggcgaccaccgaGATCGGAAGAGCACACGTCTGAACTCCAGTCACNNNNNNgcacaaaaggaaactcaccct and reverse primer: caagcagaagacggcatacgaCGACTCGGTGCCACTTTTTC, which includes necessary adaptor and indexing sequences. “N” refers to the variable index sequence. PCR parameters were 98 °C for 30 seconds followed by 26 cycles of 98 °C for 15 seconds, 62.5 °C for 15 seconds, 72 °C for 20 seconds, and ending at 72 °C for 6 minutes. Samples were then ramped down to 4 °C and held. The resulting PCR products from multiple reactions were pooled, and unincorporated primers were removed using the GeneRead Size Selection Kit (Qiagen). Quality and purity of the PCR product were assessed by bioanalyzer (Agilent), and sequencing was performed on an Illumina HiSeq 2500 as described [17]. Informatic analysis of the raw reads was performed as described [17].

### Growth screen

To examine the effect of individual gene knockdown on cell growth, sgRNA from the CRISPRi sublibrary for mitochondria, motility, and trafficking represented in a K562 cell population were compared before and after 7 days of growth. Six million cells were collected before and after 7 days of culture in medium containing different metabolic substrates, maintained at a cell density between 250 k/mL and 1 M/mL. Medium consisted of RPMI-1640 (includes 11 mM glucose) and 50 μg/mL uridine, along with 2.5 mM pyruvate (basal), 20 mM 2DG and 2.5 mM pyruvate (respiratory), or 1 μM oligomycin (glycolytic). Growth phenotypes were calculated similar to ATP phenotype, as the average of the highest 3 log2 ratios for each gene, post-7 days’ growth versus pregrowth.

### Luciferase assay

Luciferase measurements were performed using the CellTiterGlo 2.0 kit (Promega, Madison, WI), and luminescence was measured on a Biotek H4 plate reader.

### Mitochondrial-enriched CRISPRi library screening with CoQ10

K562 cells transfected with dCas9-KRAB, ATP FRET sensor, and CRISPRi pooled library were cultured with 50 μM CoQ10 or N,N-dimethylformamide (DMF) vehicle alone for 5 days. The cells were maintained at a cell density between 250 k/mL and 1 M/mL prior to sorting 6-million–cell high- and low-ATP fractions under acute respiratory conditions as described above. Genomic DNA was processed and sequenced for sgRNA quantification as described. Three CoQ10-treated replicates and 2 vehicle-treated replicates were performed. ATP phenotype rescue was calculated as the difference between each CoQ10-treated gene’s ATP phenotype and its average vehicle-treated ATP phenotype. A gene was determined to have strong rescue if it had either a *t* test *p*-value of less than 0.05 and rescue larger than 1 SD from the average rescue of non-targeting guides, or a *t* test *p*-value of less than 0.1 and a rescue larger than 2 SDs from the average rescue of non-targeting guides.

### Mini-library and CoQ10 ATP phenotype rescue

Individual sgRNAs were selected to create a small library that could be screened more rapidly with analysis of fewer cells. From the primary screen, 1 to 3 sgRNAs/gene were selected based on robust decreased ATP phenotype (see S4 Table for list of sgRNAs and genes). This small library consisted of 161 sgRNAs targeting 68 genes and 20 non-targeting sgRNAs. sgRNAs were cloned by synthesizing 2 oligonucleotides with complementary sequences per sgRNA with necessary overhangs, annealing the oligonucleotides, and individually ligating into the sgRNA lentiviral backbone plasmid as used by Gilbert and colleagues (2014). Sequences were checked by Sanger sequencing of individual clones. DNA from individual sgRNAs was then pooled in approximately equal amounts, and the presence of all sgRNAs was confirmed by deep sequencing of the pooled plasmids after PCR amplification of the sgRNA regions. Lentivirus was created, and cells were selected for integration of the sgRNA-bearing sequence as in the primary screen.

To identify genetic causes of mitochondrial dysfunction responsive to bioenergetic therapy, K562 cells containing 1 sgRNA from the above small library were screened as in the primary screen in respiration-only substrates after they were grown in culture media containing either no supplement or 50 μM CoQ10 for at least 72 hours. The cells were maintained at a cell density between 250 k/mL and 1 M/mL and then sorted by flow cytometry into high- and low-ATP fractions of 1 million cells each under acute treatment of respiration-only substrate. Genomic DNA was processed and sequenced for sgRNA quantification as described. ATP phenotype rescue was calculated as the difference between each untreated gene’s ATP phenotype and its ATP phenotype when cultured with 50 μM CoQ10.

### Sequences of individual sgRNAs used in experiments

Below are the sequences of individual sgRNA used in experiments.

Control 1 (non-targeting) GGCCGTGGTACTGTAAAGA

Control 2 (non-targeting) GAGGGAGCTTGGTCCAACCCC

*MRPL10* GGCTTCCGTCCATTCTTCCGG

*METTL17* GGCGTTGGGACTGAGGGTCAC

*PDSS2* GTGCCGCGGGAAACAAACCAG

### Determination of intracellular CoQ10 levels and redox state

K562 cell lines with single-gene knockdown were cultured for 5 days with or without 50 μM CoQ10. One million cells of each line were pelleted, followed by flash freezing in liquid nitrogen. CoQ10 and ubiquinol were isolated from cell pellets by 1-propanol extraction, and the masses of each species per cell pellet were quantified by HPLC and mass spectrometry by the Vanderbilt Neurochemistry Core. Total CoQ10 pool size was calculated as the sum of CoQ10 and ubiquinol masses per cell pellet, and percent oxidation was calculated as the mass of CoQ10 divided by the total mass of CoQ10 and ubiquinol.

### Gene function analysis

Gene functional categories within the mitochondria were assigned based on manual evaluation of Online Mendelian Inheritance in Man (omim.org) and GeneCards (genecards.org).

### Respiration and glycolysis

Respiratory and glycolytic rates in K562 cell lines were measured with the Seahorse Extracellular Flux (XF) Analyzer 96-well plate reader. Cells were seeded at 150,000 cells per well in Seahorse assay medium (Agilent Technologies numer 103335–100), supplemented with 10 mM pyruvate, in a 96-well culture plate precoated with 22.4 μg/mL Cell-Tak (Corning number CB40240). Cells were then adhered to the plate surface by centrifugation at 200 g for 5 minutes, followed by a 40-minute incubation at 37 °C without CO_2_.

Respiration and glycolysis were simultaneously measured based on oxygen consumption rates (OCRs) and extracellular acidification consumption rate (ECAR), respectively. OCR and ECAR were measured 3 times before injection and 3 times after sequential injection of oligomycin (1 μM) or FCCP (1 μM) and rotenone (1 μM). The measurements at each time point were normalized to the value of the first time point on a well-by-well basis.

### qRT-PCR

qRT-PCR gene relative expression quantifications were performed using 7900HT Fast Real-Time PCR System (Applied Biosystem) and using FAM-MGB TaqMan Gene Expression Assays (ThermoFisher, assay ID: Hs01680112_mH—COX11; Hs01550960_g1—COX16; Hs01043634_m1—ATP5MPL; Hs00372008_m1—PDSS1; Hs01047689_m1—PDSS2; and Hs00204417_m1—NDUFA8), together with VIC-MGB human ACTB (β-actin) (ThermoFisher number 4326315E) as endogenous control. cDNAs and PCR reactions were prepared according to the protocol for Cells-to-C_T_ kit (ThermoFisher number AM1728), using the standard reverse transcription cycle (37 °C for 6 minutes, inactivation at 95 °C for 5 minutes, hold at 4 °C), and qRT-PCR conditions (UDG Incubation: 50 °C for 2 minutes; enzyme activation: 95 °C for 10 minutes; PCR cycle: 95 °C for 15 seconds, 60 °C for 1 minute—repeat 40 cycles). All reactions were performed in a 384-well plate, in duplicate and from 2 to 3 independent experiments. C_T_ (threshold cycle) values of each gene were averaged and calculated relative to C_T_ values of β-actin using the the 2^−ΔΔCT^ method [53].

### Western blot analysis

Protein levels were assessed by western blotting. HCC827 cells were collected and lysed in lysis buffer (1% sodium deoxycholate, 0.1% SDS, 25 mM Tris-HCl, 150 μM NaCl, 1% TritonX, 0.2 mM EDTA, 2% NaF, 1% sodium vanadate, 4% protease inhibitor, and 0.1% calyculin A). Equal amounts of proteins (20 μg/lane) were separated by SDS-PAGE and transferred onto polyvinylidene fluoride (PVDF) membranes (Trans-BlotTurbo Transfer Pack, Bio-Rad). K562 cells were collected and lysed in RIPA buffer (ThermoFisher number 89900). Equal amounts of proteins (40 μg/lane) were separated by SDS-PAGE and transferred onto nitrocellulose membranes using iBlot 2 Dry Blotting System (ThermoFisher number IB23001 and IB21001). Primary antibodies used recognized human anti-METT11D1 antibody (ab103318, 1:1000; Abcam), β-actin mAbs (number 3700, 1:1500; Cell Signaling Technology), human anti-β-actin antibody (ab20272, 1:2000; Abcam), anti-NDUFS4 antibody (ab55540, 1:1500; Abcam), and anti-Tom20 antibody (sc-11415, 1:500; Santa Cruz Biotechnology). An anti-mouse IgG, HRP-linked secondary antibody (number 7076, 1:3000; Cell Signaling Technology) was used for HCC827 cells. Blots were developed using the Amersham ECL Western Blotting Detection Reagents (GE Healthcare), and bands were visualized using the ChemiDoc Touch Imaging System (Bio-Rad). IRDye 800CW Goat anti-Mouse IgG and IRDye 680RD Goat anti-Rabbit IgG secondary antibodies (1:10000; LI-COR Biosciences) were used for K562 cells. Blots were visualized using the Odyssey infrared imaging system and analyzed using Image Studio Lite Software (LI-COR Biosciences).

### Cell cycle and annexin V analysis

Cell cycle was assessed using propidium Iodide staining and FACS as described [54]. HCC827 cells were seeded in quadruplicates in 6-well plates and incubated overnight. Cells were treated with 0, 10, or 20 mM of 2DG in medium supplemented with 1 μg/mL puromycin, 1.5 mM pyruvate, and 0.05 mg/mL uridine. Samples were collected after 24 hours and fixed in 70% ethanol. Fixed cells were treated with RNase and PI for 2 hours and analyzed by flow cytometry (BD Biosciences FACS Verse). The FITC Annexin V apoptosis detection kit (catalog number 556547; Becton Dickinson) was used per manufacturer’s instructions, and cells were analyzed by flow cytometry (BD Biosciences FACS Verse). The results were analyzed using FlowJo V.10.1 software (FlowJo, LLC, Ashland, OR).

### Trypan blue viability staining

Cells were plated at a density of 2,000 cells/well in 6-well plates, and 24 hours after plating, medium was changed to control or 2DG-containing media. Cells were cultured under standard conditions over 48 hours, at which point cells were trypsinized, centrifuged, washed with PBS, and resuspended in 1 mL of PBS. The collected cell suspension for each replicate was stained with 0.4% (w/v) trypan blue (catalog number 15250061; Thermo Fisher Scientific) in 1:1 ratio, and cells were counted using a hemocytometer.

### Statistics

All statistical analyses—including *n*, what *n* represents, description of error bars, statistical tests used, and level of significance—are described in the figure legends. FACS analyses were graphed with box plots, with the top and bottom of the box indicating the interquartile range, the whiskers indicating the 5th and 95th percentiles, and the center line in the box indicating the median. One- or two-way ANOVA followed by multiple comparisons tests were used to compare ATP levels, assessed with either luciferase or the ATP FRET sensor-based assays, with *p* < 0.05 considered significant. Correlation analyses were performed with linear regression and determined to have non-0 slopes via F-test. For the respiratory condition, we defined a hit as a gene with 2 or more sgRNAs >2 SDs beyond the phenotype of the non-targeting sgRNAs after averaging the 3 repetitions of the respiratory-condition screen. We removed hits that showed a phenotype by these same criteria in the same direction with the Dead mutant sensor. For the glycolytic and basal conditions, which had narrower distributions of the non-targeting sgRNAs, we defined a hit as having 3 or more sgRNAs >2 SDs beyond the average phenotype of the non-targeting sgRNAs, and we removed hits with 2 or more sgRNAs >2 SDs beyond non-targeting sgRNAs with the Dead sensor. Phenotypes (mean of best 3 sgRNAs averaged over all repetitions) for all screened genes in all conditions are provided in S1 Table. GraphPad Prism and FlowJo were used to generate graphs, and GraphPad Prism was used for statistical analyses.

Software for processing and analysis of sequencing reads from pooled library was the same as that used by Gilbert and colleagues (2014). Gene names used were HUGO Gene Nomenclature Committee (HGNC)-approved symbols (https://www.genenames.org). For the ATP FRET-based screen, we used the “rho” calculation comparing the high and low FRET pools as described. We required at least 50 reads in 1 of the 2 pools for an sgRNA to be included in analysis. Gene phenotypes were calculated as the mean of the 3 sgRNAs with the strongest phenotype. No growth value was used in the FACS data analysis because this was not a growth screen. Phenotypes (average of strongest sgRNAs) for all genes screened in all conditions are provided in S1 Table. Sequences for all sgRNAs were published by Gilbert and colleagues (2014).

## Supporting information

S1 Fig Clover-mApple ATP FRET sensor measures relative changes in ATP independent of sensor concentration. (A) Cells expressing either the CFP-Venus ATP FRET sensor (blue) or the corresponding CFP-Venus Dead sensor (green) were subjected to flow cytometry. The graph shows the relative intensity of the FRET channel (y-axis) as a function of the donor (CFP) fluorescence. Approximately 1,200 cells per group; experiment repeated twice with similar results. (B) Cells expressing the CFP-Venus ATP FRET sensor (blue, untreated) were treated with 5 μM oligomycin and 10 mM 2DG for 30 minutes (purple) to block ATP synthesis prior to flow cytometry. Blocking ATP synthesis markedly decreased the ATP FRET signal as a function of the donor concentration. A total of 1,200 cells per group; experiment repeated twice with similar results. (C) FRET/Donor ratio (x-axis) of the CFP-Venus ATP FRET sensor as a function of the acceptor fluorescence (y-axis), used as a surrogate for sensor expression level. The FRET/Donor ratio depends heavily on the sensor concentration, especially at lower expression levels. (D) Fluorescent microcopy image of the Clover-mApple sensor in K562 cells shows cytoplasmic localization of both fluorophores. (E) FRET versus donor fluorescence of the Clover-mApple ATP FRET sensor (red), and the corresponding Clover-mApple Dead sensor (orange), were analyzed by flow cytometry. Approximately 3,400 cells per group; experiment repeated twice with similar results. (F) Cells expressing the Clover-mApple ATP FRET sensor (red, untreated) were treated with 5 μM oligomycin and 10 mM 2DG for 30 minutes (purple) to block ATP synthesis prior to flow cytometry. Blocking ATP synthesis markedly decreases the ATP FRET signal as a function of the donor concentration. Approximately 3,400 cells per group; experiment repeated twice with similar results. (G) FRET/Donor ratio (x-axis) of the Clover-mApple ATP FRET sensor (y-axis) as a function of the acceptor fluorescence shows that the FRET/Donor ratio is independent of the sensor expression level. (H) FRET signal of cell lysates prepared from COS cells expressing either the CFP-Venus ATP FRET sensor (blue) or the CFP-Venus Dead FRET sensor (green) and incubated with increasing concentrations of ATP. The live sensor was responsive to ATP concentrations up to approximately 3 mM, a significantly lower dynamic range than the Clover-mApple ATP FRET sensor (see Fig 1B). Data show mean ± SD (bars obscured by points); *n* = 2 wells/group. Further information about this figure can be found in S2 Data. 2DG, 2-deoxyglucose; CFP, cyan fluorescent protein; COS, CV-1 (simian) in origin, and carrying the SV40 genetic material; FRET, fluorescence resonance energy transfer.
(TIF)

S2 Fig Change in FRET with clover-mApple dead FRET sensor and luciferase measurements in glycolytic conditions. (A) Replication of stable FRET change in the respiratory condition. K562 cells expressing the Clover-mApple ATP sensor were treated as described for Fig 2B. The repetition shows a similar decrease in ATP stable for 60 minutes in the respiratory condition (blue box and whiskers) and complete loss of ATP if oxidative phosphorylation is also blocked (red box and whiskers). (B) Time course of FRET change by flow cytometry after maximal inhibition of both glycolysis and respiration (10 mM 2DG and 5μM oligomycin; red box and whisker plots; line = median; box = 25th–75th percentile; whisker = 5th–95th percentile) or no drug treatment (black box and whisker plots). *p <* 0.0001 versus both control at each time point after start by two-way ANOVA with Sidak multiple comparisons test; *n* = 11,721–18,714 cells sorted per group. (C) Time course of ATP decrease by luciferase assay after maximal inhibition of both glycolysis and respiration (10 mM 2DG and 5 μM oligomycin, red lines). ATP levels of cells expressing Clover-mApple ATP (solid lines) and Clover-mApple Dead (dotted lines) sensors decrease similarly versus no drug treatment (black lines). Data show mean ± SEM; *n* = 4 independent experiments, with each experiment a compilation of 2 samples. (D) Time course of ATP decline following incubation of cells with a respiratory inhibitor (5 μM oligomycin) in 2 mM glucose to force reliance on glycolysis for ATP (“glycolytic” conditions; note that 3 mM 2DG was also added such that ATP levels decrease below baseline), or when both respiration and glycolysis were blocked (10 mM 2DG and 5 μM oligomycin) to prevent all ATP production. Note that the data in panels A and D were obtained as part of the same experiment but are presented as separate panels for clarity and flow of presentation. The same data for the No Treatment and Glycolysis and Respiration Blocked groups is shown in both panels for reference. ATP was measured by FRET with the Clover-mApple ATP sensor using flow cytometry (box and whisker plots). Glycolytic conditions produce a small drop in ATP level that is stable through at least 75 minutes. *p <* 0.0001 versus both the control and blocked glycolysis/respiration blocked groups at each time point after start by two-way ANOVA with Tukey multiple comparisons test; *n* = 6,056–18,647 cells per group. Further information about this figure can be found in S2 Data. 2DG, 2-deoxyglucose; FRET, fluorescence resonance energy transfer.
(TIF)

S3 Fig Individual sgRNA phenotypes for select genes. Selected genes considered “hits” by reducing ATP in the respiratory condition when knocked down were graphed with each sgRNA separately from each of the 3 experimental repetitions (represented as blue, orange, and magenta). Each gene was targeted by 10 distinct sgRNAs, but some sgRNAs failed to yield sufficient read counts to be included and thus were not analyzed. Compared with a random simulated gene composed of non-targeting sgRNAs (lower right), genes identified as hits had multiple sgRNAs that decreased ATP in multiple repetitions. Not all sgRNAs were expected to be active as described by Gilbert and colleagues (2014). sgRNA, single guide RNA.
(TIF)

S4 Fig CRISPRi has minimal impact on ATP levels under basal conditions during which both respiration and glycolysis are active. Cells expressing an sgRNA from the mitochondrial-gene–enriched CRISPRi sublibrary with 10 sgRNAs/gene and the Clover-mApple-ATP were placed under “basal” conditions in which cells have uninhibited ATP synthesis from aerobic respiration and glycolysis (pyruvate and glucose as substrates, no metabolic inhibitors), and the cells were FACS sorted, based on ATP concentration. The abundance of each sgRNA in the high- and low-FRET fractions was determined by deep sequencing, and the relative enrichment of each sgRNA in the high- versus low-ATP fraction was determined. The graph shows the fold-enrichment of individual genes in the high- versus low-ATP quartiles of FRET (y-axis), with each point representing the mean enrichment of the 3 sgRNAs with the largest fold-enrichment magnitudes, in basal conditions without metabolic inhibition. The genes are grouped by general function, including motility, trafficking, “mito protein synth,” “resp chain,” and “other mito.” CRISPRi against most genes has little impact on ATP levels in this metabolic condition. CRISPRi, clustered regularly interspaced short palindromic repeats interference; FACS, fluorescence-activated cell sorting; FRET, fluorescence resonance energy transfer; other mito, other mitochondrial conditions; mito protein synth, mitochondrial protein synthesis; resp chain, respiratory chain; sgRNA, single guide RNA.
(TIF)

S5 Fig Validation of gene knockdown. qRT-PCR of gene knockdown for Fig 6. Relative fold-changes in transcription of K562 knockdown lines, analyzed by RT-qPCR. Data represent the fold-change in the knockdown gene’s transcript relative to β-actin transcript level. Data show SEM; *n* = 2–4 experiments per cell line; 2 wells per group in each experiment. *p < 0.05; **p < 0.01 versus control by one-way ANOVA with Dunnett multiple comparisons test. Further information about this figure can be found in S2 Data. qRT-PCR, quantitative real-time reverse transcription PCR.
(TIF)

S6 Fig Selected low-ATP hits do not significantly decrease mitochondrial content. Representative western blot data show that all K562 knockdown lines tested have similar mitochondrial content, as assessed by the levels of Tom20 (outer mitochondrial membrane protein) and NDUFS4 (complex I subunit, mitochondrial inner membrane). Bar graph shows quantification of Tom20 and NDUFS4 relative to β-actin pixel intensity by western blot. *n* = 3 experiments; *N* = 1 sample per group. Data show mean ± SEM. Further information about this figure can be found in S2 Data. NS, not significant by one-way ANOVA with Dunnett multiple comparisons test.
(TIF)

S7 Fig ATP from mitochondria promotes but is not always required for cell growth. K562 cells expressing the CRISPRi library were grown in media favoring respiration only (panel A and D), glycolysis only (panel C and F), or under basal conditions that allow both respiration and glycolysis (panel B and E), and the genes enriched and depleted in each metabolic condition were determined. This growth screen was performed in duplicate, and the growth phenotype of each gene knockdown was plotted here against its respective average ATP phenotype for each metabolic condition in each replicate. The compilation of these runs under respiratory conditions (panel A and D) is shown in Fig 7C, while the compilation under glycolytic conditions (panel C and F) is shown in Fig 7D. (A) In the first replicate, under respiratory conditions, when GROWTH_resp_ was compared to ATP_resp_, MRP (purple squares) and MPI (orange diamonds) genes had strong positive slopes (0.323 ± 0.125 [*p* = 0.0096] and 0.599 ± 0.157 [p = 0.0008], respectively, by F-test), and CRP genes (green triangles) did not (−0.0634 ± 0.0462 [*p* = 0.1740]). (B) In basal conditions, when GROWTH_bas_ was compared to ATP_basal_, no subset was different from 0 (MRP slope = −0.0625 ± 0.0519 [*p* = 0.2316]; CRP slope = −0.0113 ± 0.0916 [*p* = 0.902]; and MPI slope = −0.12 ± 0.0908 [*p* = 0.199]). (C) Under glycolytic conditions, when GROWTH_glyc_ was compared to ATP_glyc_, MRP had an increased slope (0.163 ± 0.0411 [*p* = 0.0001]), while MPI and CRP did not (−0.127 ± 0.0745 [*p* = 0.1] and 0.00181 ± 0.0589 [*p* = 0.976], respectively). (D) In the second replicate, under respiratory conditions, when GROWTH_resp_ was compared to ATP_resp_, MRP (purple squares) and MPI (orange diamonds) genes had strong positive slopes (0.664 ± 0.092 [*p* < 0.0001] and 0.467 ± 0.238 [*p* = 0.0626], respectively, by F-test), and CRP genes (green triangles) did not (0.013 ± 0.0910 [*p* = 0.8879]). (E) In basal conditions, when GROWTH_bas_ was compared to ATP_basal_, no subset was different from 0 (MRP slope = 0.103 ± 0.0531 [*p* = 0.0576]; CRP slope = −0.14 ± 0.0941 [*p* = 0.143]; and MPI slope = 0.136 ± 0.0878 [*p* = 0.135]). (F) Under glycolytic conditions, when GROWTH_glyc_ was compared to ATP_glyc_, MRP had a non-0 slope (−0.161 ± 0.056 [*p* = 0.0056]), while MPI and CRP did not (−0.207 ± 0.127 [*p* = 0.118] and −0.0652 ± 0.0958 [*p* = 0.500], respectively). Further information about this figure can be found in S2 Data. ATP_basal_, ATP levels under basal conditions; ATP_resp_, ATP levels under respiratory conditions; CRISPRi, clustered regularly interspaced short palindromic repeats interference; CRP, cytosolic ribosomal protein; GROWTH_glyc_, growth under glycolytic conditions; GROWTH_resp_, growth under respiratory conditions; MPI, mitochondrial protein import; MRP, mitochondrial ribosomal protein.
(TIF)

S8 Fig *METTL17* silencing reduces cell proliferation in HCC827 human lung cancer cells. (A) METTL17 knockdown in Fig 8. Lentivirus expressing negative-control sgRNA or sgRNA targeting METTL17 was transduced into HCC827 cells expressing dCas9. After growth in 1 ug/mL puromycin for 1 week, total METTL17 protein levels were significantly lower in sgRNA METTL17-expressing cells than negative controls. (B) Cells expressing either negative-control sgRNA or sgRNA targeting *METTL17* were compared by trypan blue viability assay to quantify numbers of cells growing 48 hours after plating identical numbers of cells. *METTL17* silencing is associated with significantly reduced cell proliferation compared with non-targeting control. A total of 20 mM 2DG exposure for 24 hours significantly and similarly reduced numbers of both control and *METTL17*-silenced HCC827 cells (mean +/− SEM shown; *t* test, **p < 0.001; ***p < 0.0001). (C) Unsynchronized cells stained with PI 24 hours after exposure to control media or media with 10 or 20 mM 2DG. Cells were analyzed by FACS, and G1 and G2 fractions were quantified. Exposure to 2DG caused an increase in the G1 fraction for both sgRNA negative controls but not *METTL17*. Data show mean ± SEM; *n* = 4 samples/group; 10,000 sorted per group. ***p < 0.001 versus control by two-way ANOVA with Tukey multiple comparisons test. (D) Unsynchronized cells stained with Annexin V-FITC 24 hours after exposure to control media or media with 20 mM 2DG. Cells treated with H_2_O_2_ for 30 minutes were included as a positive control for death induction. Cells were analyzed by FACS and percentage of FITC-positive cells were quantified. Data show mean ± SEM. Further information about this figure can be found in S2 Data. 2DG, 2-deoxyglucose; FACS, fluorescence-assisted cell sorting; PI, propidium iodide; sgRNA, single guide RNA.
(TIF)

S1 Table Phenotypes of all genes in all conditions from the ATP FRET screen. FRET, fluorescence resonance energy transfer.
(XLSX)

S2 Table Phenotypes for all individual sgRNAs in the respiratory condition. sgRNA, single guide RNA.
(XLSX)

S3 Table Genes identified as hits in the respiratory, glycolytic, and basal conditions. (XLSX)

S4 Table Sequences of sgRNAs used in CoQ10 rescue experiments and experiments with individual sgRNAs. CoQ10, coenzyme Q10; sgRNA, single guide RNA.
(XLSX)

S5 Table Phenotypes for all genes in the CoQ10 rescue experiments. CoQ10, coenzyme Q10.
(XLSX)

S1 Data Raw data used for quantification in this work. (XLSX)

S2 Data Raw data used for quantification for supporting information in this work. (XLSX)

## Abbreviations

2DG: 2-deoxyglucose
ATPbasal: ATP levels under basal conditions
ATPgly: ATP levels under glycolytic conditions
ATPresp: ATP levels under respiratory conditons
CFP: cyan fluorescent protein
CoQ10: coenzyme Q10
COS: CV-1 (simian) in origin, and carrying the SV40 genetic material
CRISPRi: clustered regularly interspaced short palindromic repeats interference
dCas9: dead CRISPR-associated protein 9
ECAR: extracellular acidification rate
FACS: fluorescence-activated cell sorting
FBS: fetal bovine serum
FCCP: carbonyl cyanide-*4*-(trifluoromethoxy)phenylhydrazone
FRET: fluorescence resonance energy transfer
GROWTH_basal_: growth under basal conditions
GROWTH_glyc_: growth under glycolytic conditions
GROWTH_resp_: growth under respiratory conditions
HGNC: HUGO Gene Nomenclature Committee
HPLC: high-performance liquid chromatography
KRAB: Kruppel-associated box
MELAS: mitochondrial encephalopathy, lactic acidosis, and stroke-like episodes
MICOS: mitochondrial contact site and cristae organizing system
mVenus: monomeric Venus
NDUFS4: NADH:ubiquinone oxidoreductase subunit S4
OCR: oxygen consumption rate
PVDF: polyvinylidene fluoride
qRT-PCR: quantitative real-time reverse transcription PCR
RNAi: RNA interference
ROS: reactive oxygen species
sgRNA: single guide RNA
shRNA: short hairpin RNA
TCA: tricarboxylic acid
Tom20: translocase of outer membrane 20 kDa subunit
