# Supplementary figures and images for: A high-throughput screen of real-time ATP levels in individual cells reveals mechanisms of energy failure
Source: PLoS Biol. 2018 Aug 27;16(8):e2004624. doi: 10.1371/journal.pbio.2004624 (PMC6110572; doi:10.1371/journal.pbio.2004624)

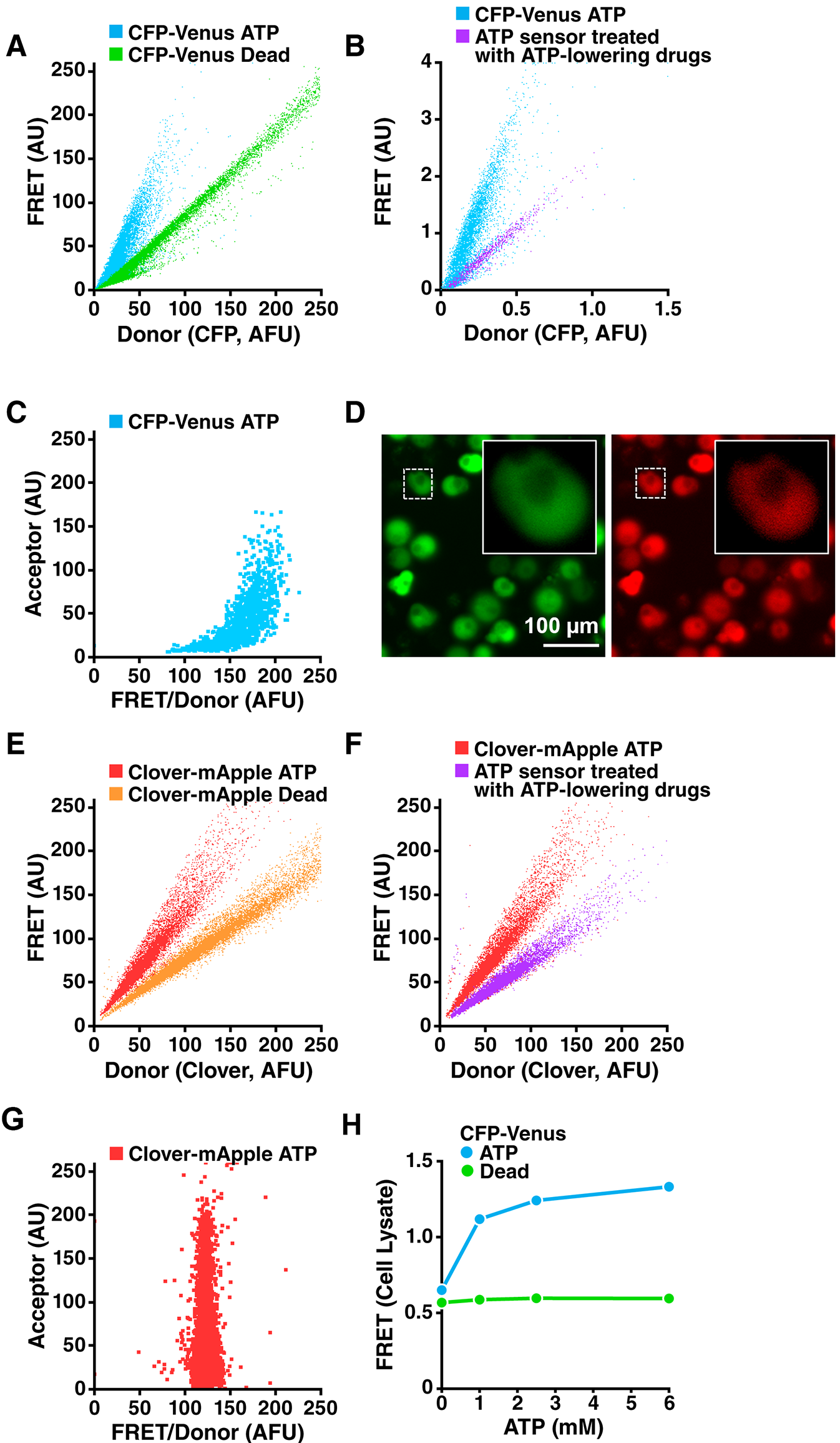

Supplement: S1 Fig — (A) Cells expressing either the CFP-Venus ATP FRET sensor (blue) or the corresponding CFP-Venus Dead sensor (green) were subjected to flow cytometry. The graph shows the relative intensity of the FRET channel (y-axis) as a function of the donor (CFP) fluorescence. Approximately 1,200 cells per group; experiment repeated twice with similar results. (B) Cells expressing the CFP-Venus ATP FRET sensor (blue, untreated) were treated with 5 μM oligomycin and 10 mM 2DG for 30 minutes (purple) to block ATP synthesis prior to flow cytometry. Blocking ATP synthesis markedly decreased the ATP FRET signal as a function of the donor concentration. A total of 1,200 cells per group; experiment repeated twice with similar results. (C) FRET/Donor ratio (x-axis) of the CFP-Venus ATP FRET sensor as a function of the acceptor fluorescence (y-axis), used as a surrogate for sensor expression level. The FRET/Donor ratio depends heavily on the sensor concentration, especially at lower expression levels. (D) Fluorescent microcopy image of the Clover-mApple sensor in K562 cells shows cytoplasmic localization of both fluorophores. (E) FRET versus donor fluorescence of the Clover-mApple ATP FRET sensor (red), and the corresponding Clover-mApple Dead sensor (orange), were analyzed by flow cytometry. Approximately 3,400 cells per group; experiment repeated twice with similar results. (F) Cells expressing the Clover-mApple ATP FRET sensor (red, untreated) were treated with 5 μM oligomycin and 10 mM 2DG for 30 minutes (purple) to block ATP synthesis prior to flow cytometry. Blocking ATP synthesis markedly decreases the ATP FRET signal as a function of the donor concentration. Approximately 3,400 cells per group; experiment repeated twice with similar results. (G) FRET/Donor ratio (x-axis) of the Clover-mApple ATP FRET sensor (y-axis) as a function of the acceptor fluorescence shows that the FRET/Donor ratio is independent of the sensor expression level. (H) FRET signal of cell lysates prepared fro [file pbio.2004624.s001.tif]

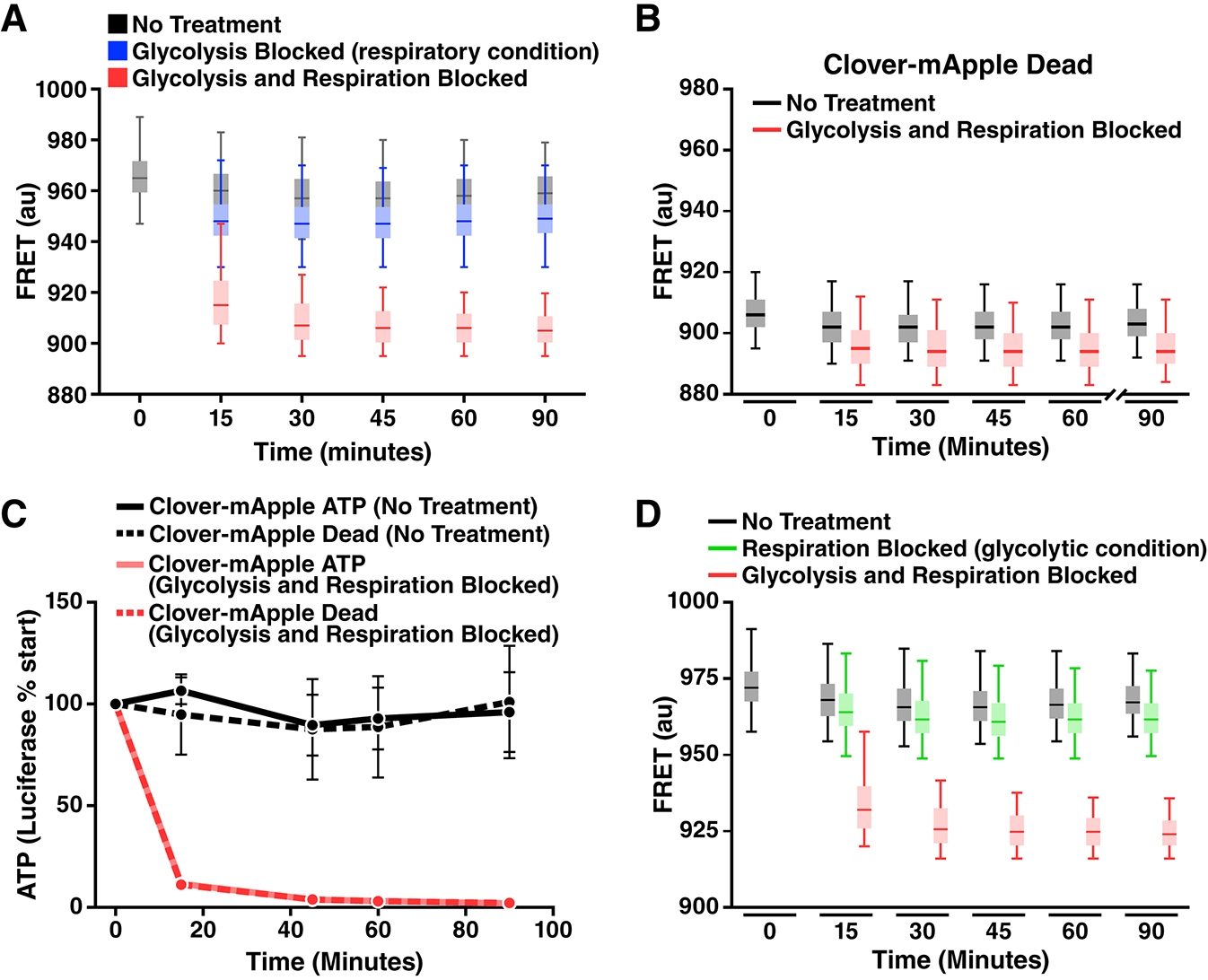

Supplement: S2 Fig — (A) Replication of stable FRET change in the respiratory condition. K562 cells expressing the Clover-mApple ATP sensor were treated as described for Fig 2B. The repetition shows a similar decrease in ATP stable for 60 minutes in the respiratory condition (blue box and whiskers) and complete loss of ATP if oxidative phosphorylation is also blocked (red box and whiskers). (B) Time course of FRET change by flow cytometry after maximal inhibition of both glycolysis and respiration (10 mM 2DG and 5μM oligomycin; red box and whisker plots; line = median; box = 25th–75th percentile; whisker = 5th–95th percentile) or no drug treatment (black box and whisker plots). p < 0.0001 versus both control at each time point after start by two-way ANOVA with Sidak multiple comparisons test; n = 11,721–18,714 cells sorted per group. (C) Time course of ATP decrease by luciferase assay after maximal inhibition of both glycolysis and respiration (10 mM 2DG and 5 μM oligomycin, red lines). ATP levels of cells expressing Clover-mApple ATP (solid lines) and Clover-mApple Dead (dotted lines) sensors decrease similarly versus no drug treatment (black lines). Data show mean ± SEM; n = 4 independent experiments, with each experiment a compilation of 2 samples. (D) Time course of ATP decline following incubation of cells with a respiratory inhibitor (5 μM oligomycin) in 2 mM glucose to force reliance on glycolysis for ATP (“glycolytic” conditions; note that 3 mM 2DG was also added such that ATP levels decrease below baseline), or when both respiration and glycolysis were blocked (10 mM 2DG and 5 μM oligomycin) to prevent all ATP production. Note that the data in panels A and D were obtained as part of the same experiment but are presented as separate panels for clarity and flow of presentation. The same data for the No Treatment and Glycolysis and Respiration Blocked groups is shown in both panels for reference. ATP was measured by FRET with the Clover-mApple ATP sensor using flow cytometry (box [file pbio.2004624.s002.tif]

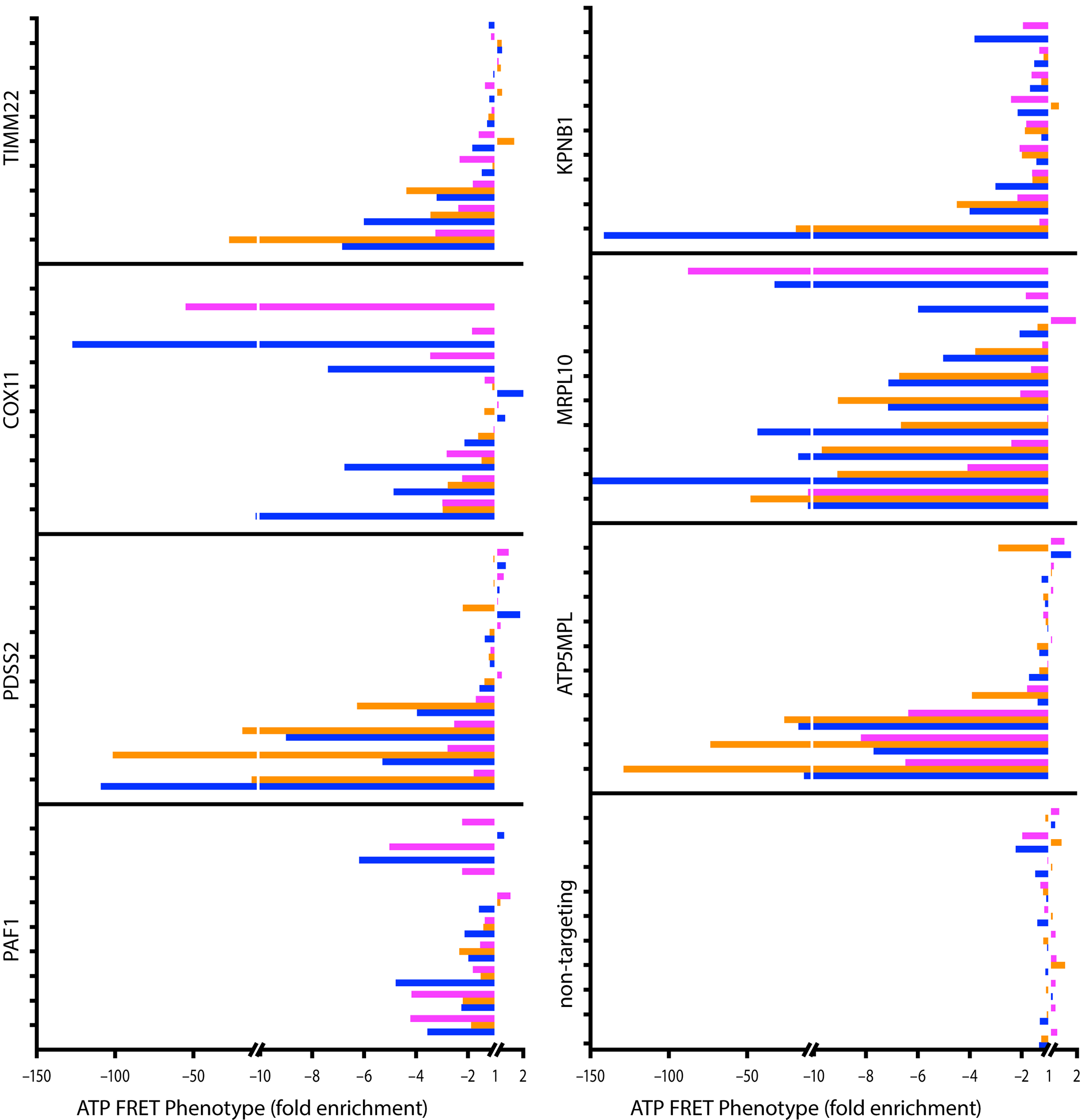

Supplement: S3 Fig — Selected genes considered “hits” by reducing ATP in the respiratory condition when knocked down were graphed with each sgRNA separately from each of the 3 experimental repetitions (represented as blue, orange, and magenta). Each gene was targeted by 10 distinct sgRNAs, but some sgRNAs failed to yield sufficient read counts to be included and thus were not analyzed. Compared with a random simulated gene composed of non-targeting sgRNAs (lower right), genes identified as hits had multiple sgRNAs that decreased ATP in multiple repetitions. Not all sgRNAs were expected to be active as described by Gilbert and colleagues (2014). sgRNA, single guide RNA. (TIF) [file pbio.2004624.s003.tif]

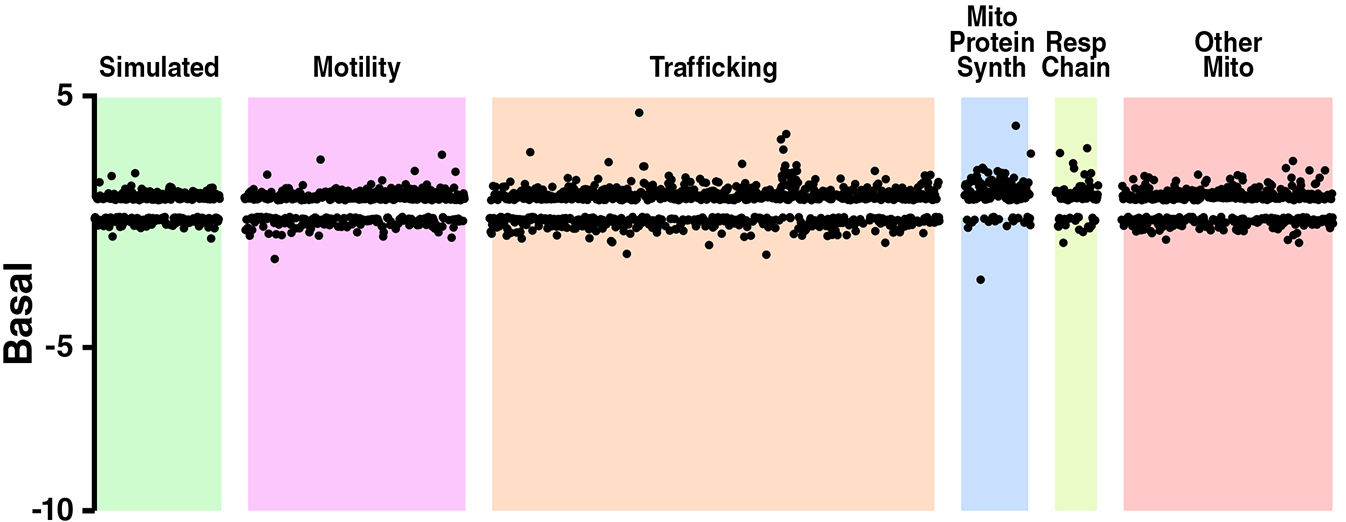

Supplement: S4 Fig — Cells expressing an sgRNA from the mitochondrial-gene–enriched CRISPRi sublibrary with 10 sgRNAs/gene and the Clover-mApple-ATP were placed under “basal” conditions in which cells have uninhibited ATP synthesis from aerobic respiration and glycolysis (pyruvate and glucose as substrates, no metabolic inhibitors), and the cells were FACS sorted, based on ATP concentration. The abundance of each sgRNA in the high- and low-FRET fractions was determined by deep sequencing, and the relative enrichment of each sgRNA in the high- versus low-ATP fraction was determined. The graph shows the fold-enrichment of individual genes in the high- versus low-ATP quartiles of FRET (y-axis), with each point representing the mean enrichment of the 3 sgRNAs with the largest fold-enrichment magnitudes, in basal conditions without metabolic inhibition. The genes are grouped by general function, including motility, trafficking, “mito protein synth,” “resp chain,” and “other mito.” CRISPRi against most genes has little impact on ATP levels in this metabolic condition. CRISPRi, clustered regularly interspaced short palindromic repeats interference; FACS, fluorescence-activated cell sorting; FRET, fluorescence resonance energy transfer; other mito, other mitochondrial conditions; mito protein synth, mitochondrial protein synthesis; resp chain, respiratory chain; sgRNA, single guide RNA. (TIF) [file pbio.2004624.s004.tif]

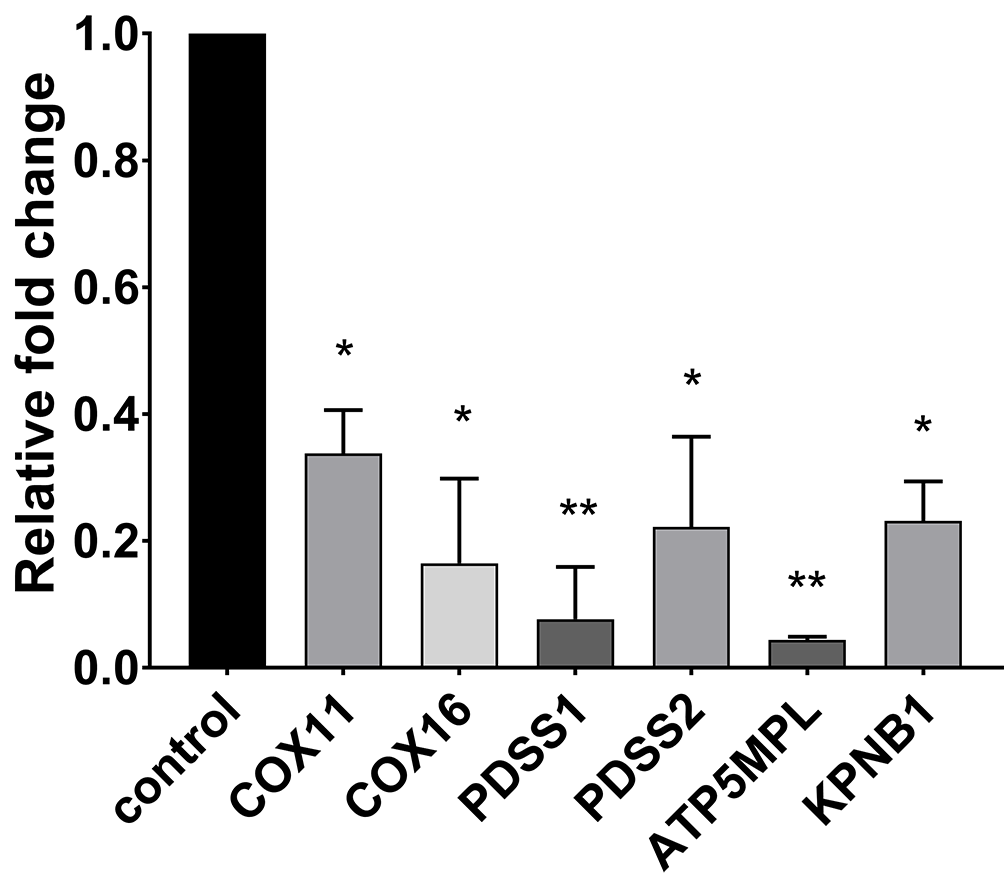

Supplement: S5 Fig — qRT-PCR of gene knockdown for Fig 6. Relative fold-changes in transcription of K562 knockdown lines, analyzed by RT-qPCR. Data represent the fold-change in the knockdown gene’s transcript relative to β-actin transcript level. Data show SEM; n = 2–4 experiments per cell line; 2 wells per group in each experiment. *p < 0.05; **p < 0.01 versus control by one-way ANOVA with Dunnett multiple comparisons test. Further information about this figure can be found in S2 Data. qRT-PCR, quantitative real-time reverse transcription PCR. (TIF) [file pbio.2004624.s005.tif]

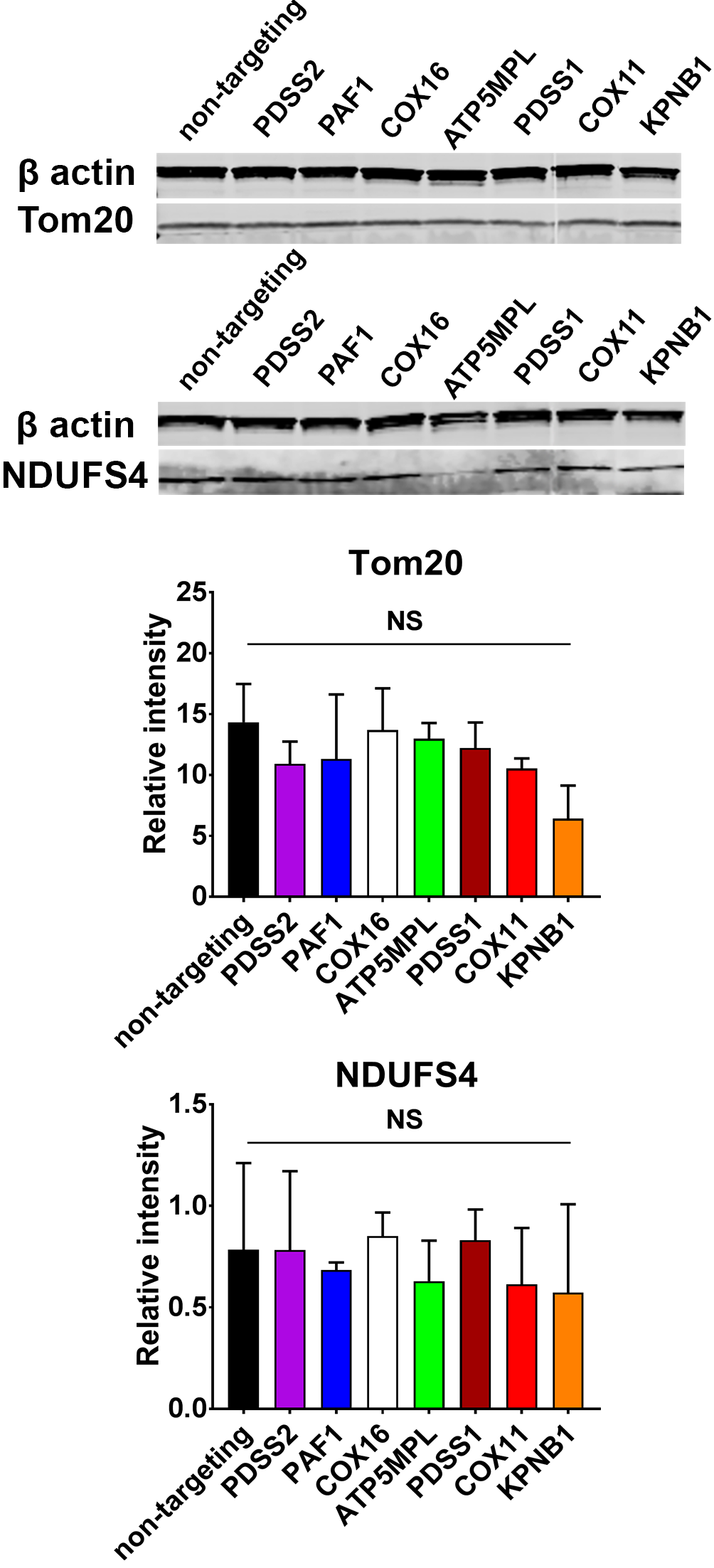

Supplement: S6 Fig — Representative western blot data show that all K562 knockdown lines tested have similar mitochondrial content, as assessed by the levels of Tom20 (outer mitochondrial membrane protein) and NDUFS4 (complex I subunit, mitochondrial inner membrane). Bar graph shows quantification of Tom20 and NDUFS4 relative to β-actin pixel intensity by western blot. n = 3 experiments; N = 1 sample per group. Data show mean ± SEM. Further information about this figure can be found in S2 Data. NS, not significant by one-way ANOVA with Dunnett multiple comparisons test. (TIF) [file pbio.2004624.s006.tif]

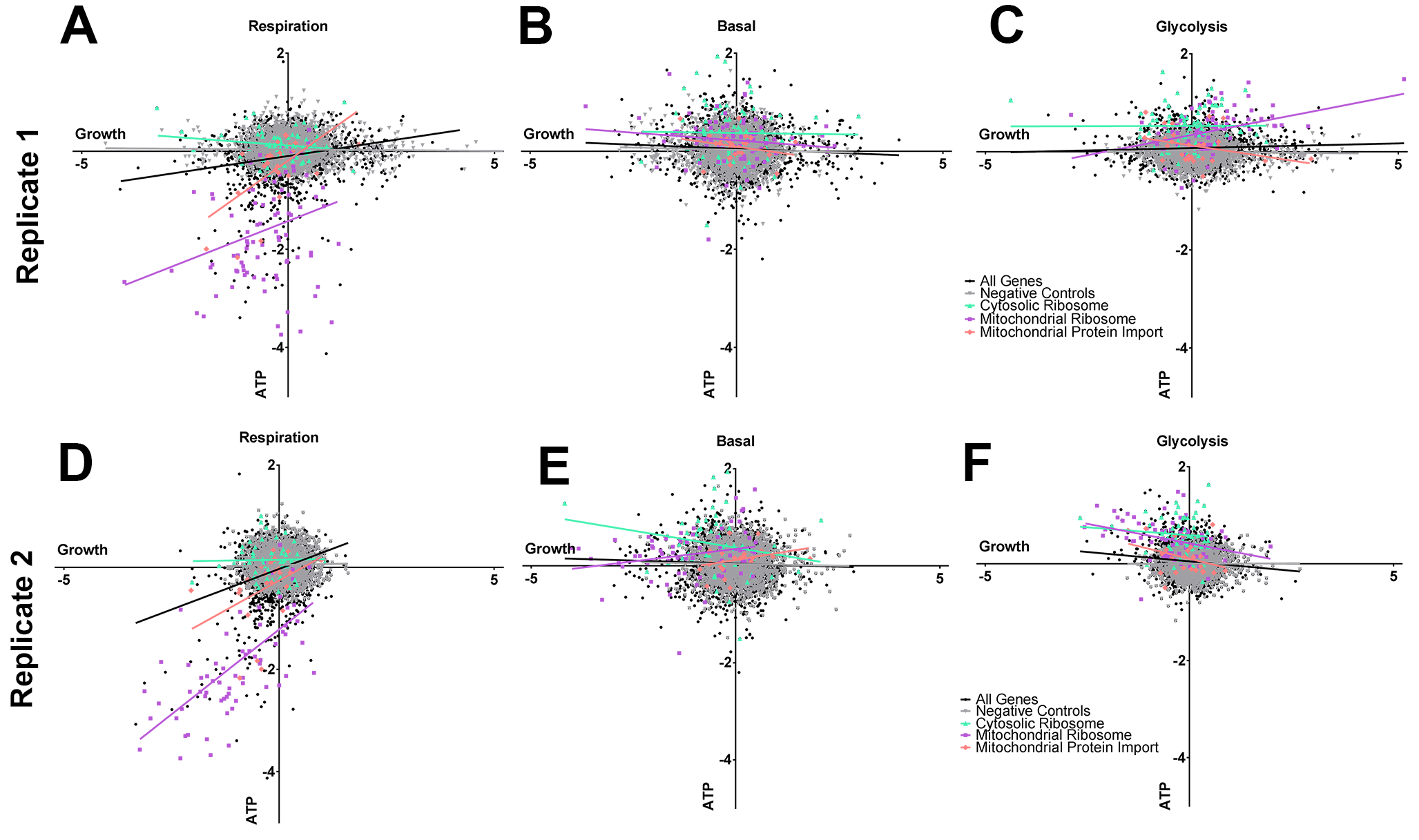

Supplement: S7 Fig — K562 cells expressing the CRISPRi library were grown in media favoring respiration only (panel A and D), glycolysis only (panel C and F), or under basal conditions that allow both respiration and glycolysis (panel B and E), and the genes enriched and depleted in each metabolic condition were determined. This growth screen was performed in duplicate, and the growth phenotype of each gene knockdown was plotted here against its respective average ATP phenotype for each metabolic condition in each replicate. The compilation of these runs under respiratory conditions (panel A and D) is shown in Fig 7C, while the compilation under glycolytic conditions (panel C and F) is shown in Fig 7D. (A) In the first replicate, under respiratory conditions, when GROWTHresp was compared to ATPresp, MRP (purple squares) and MPI (orange diamonds) genes had strong positive slopes (0.323 ± 0.125 [p = 0.0096] and 0.599 ± 0.157 [p = 0.0008], respectively, by F-test), and CRP genes (green triangles) did not (−0.0634 ± 0.0462 [p = 0.1740]). (B) In basal conditions, when GROWTHbas was compared to ATPbasal, no subset was different from 0 (MRP slope = −0.0625 ± 0.0519 [p = 0.2316]; CRP slope = −0.0113 ± 0.0916 [p = 0.902]; and MPI slope = −0.12 ± 0.0908 [p = 0.199]). (C) Under glycolytic conditions, when GROWTHglyc was compared to ATPglyc, MRP had an increased slope (0.163 ± 0.0411 [p = 0.0001]), while MPI and CRP did not (−0.127 ± 0.0745 [p = 0.1] and 0.00181 ± 0.0589 [p = 0.976], respectively). (D) In the second replicate, under respiratory conditions, when GROWTHresp was compared to ATPresp, MRP (purple squares) and MPI (orange diamonds) genes had strong positive slopes (0.664 ± 0.092 [p < 0.0001] and 0.467 ± 0.238 [p = 0.0626], respectively, by F-test), and CRP genes (green triangles) did not (0.013 ± 0.0910 [p = 0.8879]). (E) In basal conditions, when GROWTHbas was compared to ATPbasal, no subset was different from 0 (MRP slope = 0.103 ± 0.0531 [p = 0.0576]; CRP slope = −0.14 ± 0.0941 [p = 0 [file pbio.2004624.s007.tif]

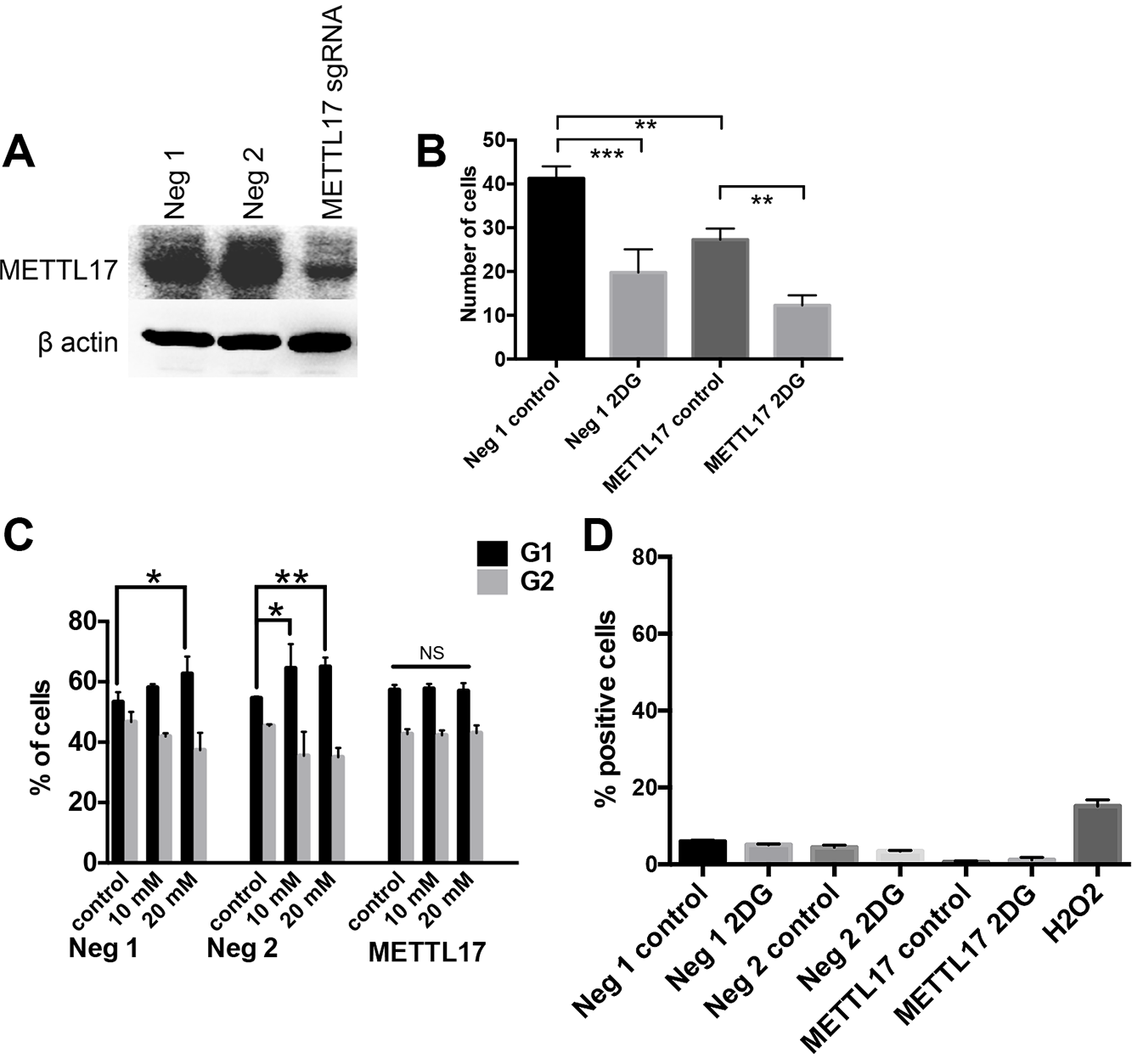

Supplement: S8 Fig — (A) METTL17 knockdown in Fig 8. Lentivirus expressing negative-control sgRNA or sgRNA targeting METTL17 was transduced into HCC827 cells expressing dCas9. After growth in 1 ug/mL puromycin for 1 week, total METTL17 protein levels were significantly lower in sgRNA METTL17-expressing cells than negative controls. (B) Cells expressing either negative-control sgRNA or sgRNA targeting METTL17 were compared by trypan blue viability assay to quantify numbers of cells growing 48 hours after plating identical numbers of cells. METTL17 silencing is associated with significantly reduced cell proliferation compared with non-targeting control. A total of 20 mM 2DG exposure for 24 hours significantly and similarly reduced numbers of both control and METTL17-silenced HCC827 cells (mean +/− SEM shown; t test, **p < 0.001; ***p < 0.0001). (C) Unsynchronized cells stained with PI 24 hours after exposure to control media or media with 10 or 20 mM 2DG. Cells were analyzed by FACS, and G1 and G2 fractions were quantified. Exposure to 2DG caused an increase in the G1 fraction for both sgRNA negative controls but not METTL17. Data show mean ± SEM; n = 4 samples/group; 10,000 sorted per group. ***p < 0.001 versus control by two-way ANOVA with Tukey multiple comparisons test. (D) Unsynchronized cells stained with Annexin V-FITC 24 hours after exposure to control media or media with 20 mM 2DG. Cells treated with H2O2 for 30 minutes were included as a positive control for death induction. Cells were analyzed by FACS and percentage of FITC-positive cells were quantified. Data show mean ± SEM. Further information about this figure can be found in S2 Data. 2DG, 2-deoxyglucose; FACS, fluorescence-assisted cell sorting; PI, propidium iodide; sgRNA, single guide RNA. (TIF) [file pbio.2004624.s008.tif]
